# Effectiveness of a Group Support Lifestyle Modification (GSLiM) Programme among Obese Adults in Workplace: A Randomised Controlled Trial

**DOI:** 10.1371/journal.pone.0160343

**Published:** 2016-08-18

**Authors:** Siti Noraida Jamal, Foong Ming Moy, Mohd Nahar Azmi Mohamed, Firdaus Mukhtar

**Affiliations:** 1 Julius Centre University of Malaya, Department of Social and Preventive Medicine, Faculty of Medicine, University Malaya, 50603 Kuala Lumpur, Malaysia; 2 Department of Sports Medicine, Faculty of Medicine, University Malaya, 50603 Kuala Lumpur, Malaysia; 3 Departments of Psychiatry, Faculty of Medicine, University Putra Malaysia, 43400 Serdang, Malaysia; University of Oxford, UNITED KINGDOM

## Abstract

**Background:**

There was an increasing trend in the prevalence of obesity and its comorbidities over the past decades in Malaysia. Effective intervention for obesity remains limited. This study aimed to compare the effectiveness of a group based lifestyle modification programme amongst obese individuals with an existing dietary counseling programme.

**Methods:**

We recruited one hundred and ninety four overweight and obese (BMI>27.5 kg/m2) employees from a local university. They were randomly allocated to either Group Support Lifestyle Modification (GSLiM) (intervention)(n = 97) or dietary counseling (comparison)(n = 97). The GSLIM activities included self monitoring, cognitive-behaviour sessions, exercise as well as dietary change advocacy, which were conducted through seminars and group sessions over 24 weeks. The comparison group was given dietary counselling once in 12 weeks. Both groups were followed up for additional 12 weeks to check for intervention effect sustenance. Anthropometric and biochemical parameters were measured at baseline, 12, 24 and 36 weeks; while dietary intake, physical activities, psychological measures and quality of life measured at baseline, 24 and 36 weeks. Data analysis was conducted using ANOVA repeated measures with intention to treat principle.

**Results:**

The participants were predominantly women with mean (standard deviation) age of 40.5 (9.3) years. A total of 19.6% of the participants in GSLiM achieved 6% weight loss compared to 4.1% in the comparison group (Risk Ratio 4.75; 95% CI: 1.68, 13.45). At 24 weeks, the retention rate was 83.5% for GSLiM and 82.5% for comparison group. GSLiM participants also achieved significant improvement in total weight self-efficacy score, negative emotions and physical discomfort subscales, MDPSS friend subscale and all domains in quality of life. Participants in the comparison group experienced reduction in negative self-thoughts.

**Conclusion:**

The GSLiM programme proved to be more effective in achieving targeted weight loss, improving weight self-efficacy, friend social support, and quality of life compared to dietary counseling.

**Trial Registration:**

Iranian Registry of Clinical Trials IRCT201104056127N1

## Introduction

Malaysia has the highest combined overweight and obese prevalence among the Southeast Asian countries in 2013 [1]. From 1996 to 2009, the prevalence of obesity in Malaysia increased by 23.7% [2], concurrent with the obesity global pandemic. High risks groups for obesity were women, married adults, those with secondary education and unemployed or homemakers [3]. Arguably, working adults who engage in sedentary work as well as those experience stress at the workplace [4,5] are also at risks of obesity.

Obesity is associated with increase mortality [6] and reduction in overall quality of life [7]. Malaysians observed an increase of mortality due to heart disease and cancers from 67% in 2008 to 73% in 2012 [8,9]. Comprehensive lifestyle modification on diet, physical activity and psychology remains as the primary intervention for obesity [10,11].

Multicomponent lifestyle modification proved successful in reducing diabetes risk [12] as well as improved cardiovascular risk factors [13]. The Diabetes Prevention Program (DPP) was a high intensity lifestyle modification programme which managed to reduce participant’s diabetic risks by 58% [12]. Yet, in real life, high intensity programme may not be applicable to all settings as it is resource intensive. This results in its translation into the Group Lifestyle Balance (GLB-DPP) implemented in clinical settings, primary care and workplace [14]. The GLB-DPP retained the core principles of the DPP such as 7% weight loss, 150 minutes of physical activity and self-monitoring using group approach with reduced sessions.

According to the Social Cognitive Theory; personal behavior, thoughts and environment reciprocate to produce action [15,16]. Factors influencing the cognitive process favoring weight loss includes high self-efficacy [17,18] and social support [19]. Meanwhile, obese individuals may have higher inclination for negative thoughts related to dysfunctional eating [20] hampering lifestyle modification progress. However, these psychological factors were seldom assessed and reported together with clinical measures as part of the lifestyle modification programme outcomes [21,22].

Workplace has been shown to be a feasible and effective setting for lifestyle modification programme for obesity prevention [23–27]. Apart from preventing short term deterioration of quality of life, moderate improvement in weight induced employee productivity [28].

In view of urgent need for obesity intervention, we implemented an adaptation of the GLB-DPP programme named Group Support Lifestyle Modification (GSLiM) in the workplace. The objective was to compare the effectiveness of the GSLiM programme with an existing one to one dietary counseling programme in the workplace. The programme was designed to create social support and improve self-efficacy. It was hypothesized that Group Support Lifestyle Modification (GSLiM) would be more effective in producing weight loss, improvement of cardiovascular risk factors, self-efficacy, social support factors as well as their quality of life.

## Methods

This was a randomised controlled trial conducted in a public university in Kuala Lumpur, Malaysia. Ethical clearance obtained from the Medical Ethics Committee, University Malaya Medical Centre on 16 March 2011 (MEC No. 841.2) and was registered with the Iranian Registry of Clinical Trial (http://www.irct.ir/) (IRCTID: IRCT201104056127N1). There was a slight delayed in trial registration as the authors waited for funding approval obtained from the University Malaya post-graduate research fund.

### Recruitment and participants

Participants were employees from a public university in Kuala Lumpur. Recruitment started soon after trial registration from May 2011 to September 2012. The trial intervention and follow up commenced from September 2011 to July 2013. Respondents gave written informed consent during recruitment and informed of random allocation into either of the treatment arms. Recruitment, data collection and intervention programme were conducted in the Sport Centre of the Faculty of Sports Science within the university.

Eligible criteria included employees of the university, Bahasa Malaysia (national language of the country) literate, with BMI of 27.5kg/m^2^ or more and able to walk briskly for at least 10 minutes without assistance. Cut off point of 27.5kg/m^2^ was used as Asians experience higher risk for type 2 diabetes, metabolic syndrome as well as cardiovascular diseases at lower BMI [29–31]. Exclusion criteria included individuals with unstable angina, congestive cardiac failure, cancer, severe pulmonary disease, psychiatry disorders e.g. substance abuse, depression, weight loss of more than 5kg in the last 6 months, pregnant or breastfeeding. Self-administered questionnaire used to assess medical history and family history for disease risks. Further assessment to rule out undiagnosed medical conditions included full blood count (FBC), renal profile (RP), fasting blood sugar (FBS) and lipid profile at recruitment phase.

**Fig 1:**
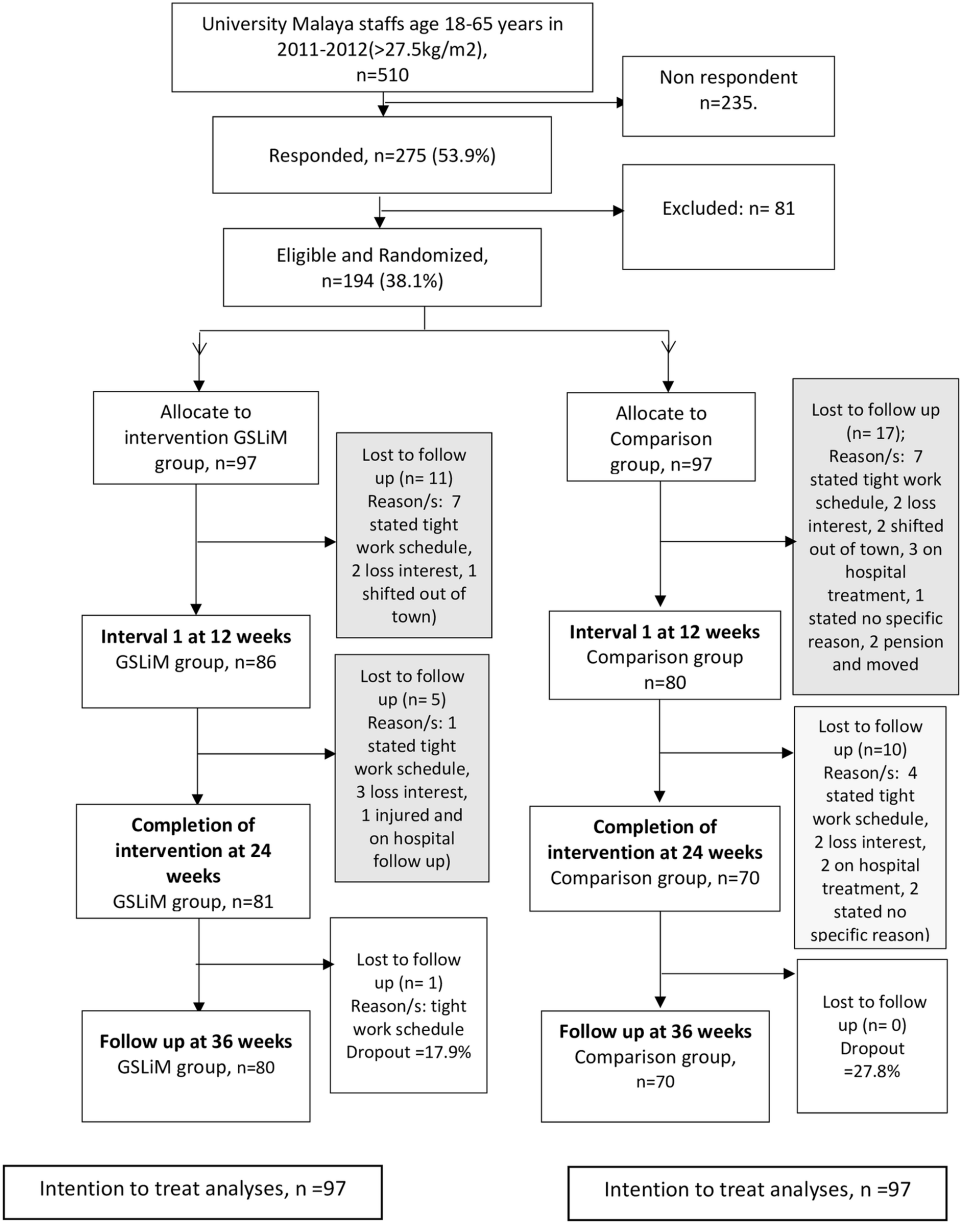
CONSORT Participants flowchart of intervention and control arm. GSLiM: Group Support Lifestyle Modification; Comparison group: Dietary counselling.

Of 510 employees’ responded to invitations via emails and flyers, 275 consented for participation and assessed for eligibility. Nineteen participants withdrew after consented, eleven did not finish screening, fifty-one were excluded and finally, 194 participants were randomised (Fig 1).

#### Randomisation

A third person (TC), who was not involved with the study conducted random allocation of participants into either Group Support Lifestyle Modification (GSLiM) or dietary counseling (comparison). Allocation of participants to GSLiM or comparison arms were assigned using opaque envelopes. This was an open label trial as both treatment provider and participants knew which arm they belonged to since the programme differed between the two groups.

### Interventions

#### GLB-DPP

The Group Lifestyle Balance (GLB-DPP) developed based on the Diabetes Prevention Program (DPP) [32] that retained the core principles of DPP. These included self-monitoring for weight, dietary and physical activity, problem solving, staying motivated, and target 7% weight loss from baseline weight. However, the programme delivery of GLB-DPP used group-approach given over twenty-two hourly sessions in three phases (core, fade frequency and support).

#### GSLiM

Permission to adopt and adapt the GLB-DPP was obtained from the author/s under the Creative Common Licence Share Alike. The GSLiM programme retained the core characteristics of GLB-DPP except a 6% target weight loss from baseline weight used instead of 7% and absence of support sessions after programme completion at 6^th^ month. The 6% target was based on an achievement of a minimum of 1% weight loss per month in view of evidence that even 5% weight loss may produce clinical improvement among obese individuals [10]. The frequency of sessions conducted in GSLiM was less compared to the GLB-DPP with ten sessions in GSLiM intervention compared to the twenty-two of GLB-DPP. However, the original twenty-two topics of the GLB-DPP were retained in GSLiM with the topics delivered through three seminars and five 90-minute sessions in two phases. The core phase (Phase 1) was run once in two weeks for three months, beginning with a two-day seminar. A fade frequency (Phase 2) started with a one-day seminar, followed by two 90-minute sessions and concluded with a half-day seminar at the end of the active intervention. Comparison of sessions between GLB-DPP and Group Support Lifestyle Modification (GSLiM) is shown in Tables 1 and 2.

**Table 1:** Comparison of Group Lifestyle Balance (GLB) with Group Support Lifestyle Modification (GSLiM) programme.

**Same aspects of GLB and GSLiM**	
Safe and appropriate intervention that incorporates nutrition, physical activity, and behaviour change. Gradual increase of 150 mins per week of physical activity and minus 500 kcal per day of dietary intake. Strong focus on use of self-monitoring tools with feedback. Initial emphasis on fat intake and calories. Primary focus on healthy food choices. Use of inexpensive food samples and incentives. Use of problem-solving techniques to address barriers to healthy eating and physical activity. Group approach.	
**Specific Adaptation to GSLiM**	
GLB	Modified GLB (GSLiM)
Goal: 7% weight loss and increase physical activity to 150 minutes/week.	Goal: 6% weight loss of baseline body weight and physical activity 150 per week.
12 weekly 1-hour sessions delivered over 12–15 weeks	0, 3 and 6 months by seminar sessions. 5 once in 2 weeks session over 12 weeks 2 monthly session over subsequent 12 weeks
Pedometer introduced during core sessions	Logging for physical activity in log book
Trainers: Dietician and exercise specialist	Trainers: Dietician and exercise specialist and psychologist
Total sessions 22 given in spread of 48 weeks	Compressed session to 10 given in 24 weeks

**Table 2:** Topic comparison between GSLiM and GLB-DPP.

GLB-DPP topic arrangement	GSLiM topic arrangements
**Core sessions: Weekly for 12 weeks**	**Core sessions: Bi- weekly (2 per month) for 12 weeks**
1. Welcome to GLB.	1. Welcome to GSLiM—Seminar overview of topics. (GLB-DPP 1) Physical activity component (overview of topics and specifics on) Stretching: The truth about flexibility (GLB-DPP session 21) Heart health (GLB-DPP session 20) Diet component: overview of topics, Tip the calorie balance (GLB-DPP 5) Psychological—overview
2. Be a fat and Calorie Detective	2. GSLiM Session 2 2.1 Fat, calorie and you (GLB-DPP session 2) 2.2 Healthy eating (GLB-DPP session 3)
3. Healthy eating	3. GSLiM Session 3 3.1 Move those muscles (GLB-DPP session 4) 3.2 Monitoring your activities (GLB-DPP 10)
4. Move those muscles	4. GSliM Session 4 4.1Negative thoughts and Weight (GLB-DPP 9 and 14) 4.2 Balance your thoughts (GLB-DPP 15)
5. Tip the calorie balance	5. GSLiM Session 5 5.1 Behavior, Environment and Lifestyle Change (GLB-DPP 6 and 17) 5.2 Make Social Cues Work for You (GLB-DPP 11)
6. Take charge of whats around you	6. GSLiM Session 6 • 6.0 Strengthen Your Exercise Programme (GLB-DPP 16)
7. Problem solving	
8. Four keys to healthy Eating out	
9. Slippery slope of Lifestyle change	
10. Jump start Your Activity Plan	
11. Make Social Cues Work for You	
12. Ways to Stay Motivated	
**Transition session (fade frequency)**	**Transition session (fade frequency)**
13. Prepare for Long Term Self Management	7. GSLiM Session 7- One day seminar.
	a. Stress management (GLB-DPP 18).
	b. Problem solving (GLB-DPP 7).
	c. Healthy eating out (GLB-DPP 8).
	d. More volume fewer calories (GLB-DPP 14).
	e. Group physical activity sessions.
14. More Volume; fewer calories	8. GSLiM Session 8. • 8.0 Standing Up for Your Health (GLB-DPP 19)
15. Balance your thoughts	9. GSLiM Session 9. • 9.0 Stay motivated (GLB-DPP 12)
16. Strengthen Your Exercise Programme	10. GSLiM session 10 (final). Looking back and looking forward (GLB-DPP 22)—Sharing achievements. Prepare long term self-management (GLB-DPP 13)
**Support sessions (variable sequence)**	**Follow up at 36 weeks**
17. Mindful eating	
18. Stress and Time Management	
19. Standing Up for Your Health	
20. Heart Health	
21. Stretching: The truth about flexibility	
22. Looking back and looking forwards	

To compensate for the lower frequency, experts in diet/nutrition, sports medicine and cognitive behaviour psychology conducted the sessions in longer duration (90 minutes). The first 30 to 40 minutes were on knowledge transfer while the subsequent duration was on practical / hands on experience such as food portion, food tasting, food weighing, aerobic exercise as well as cognitive restructuring. Bahasa Malaysia language (Malaysian national language) was used to deliver the programme. The summary of programme structure, sessions and contents are presented in Table 3.

**Table 3:** Summary of Programme Structure, Sessions and Contents.

Session	Approach	Title and Contents
**1**	Seminar	**Introduction to the programme**
		Foreword on the Group Support Lifestyle Modification (GSLiM) programme configuration and sessions. Highlight energy balance and the concept of self-monitoring. Develop allegiance to the programme.
		Set up goals for: 6% weight loss, and physical activity of 150 minutes per week. Daily calorie requirement (DCR) minus 500 for dietary intake to be worked out through the 2 step Harris Benedict equation. Foreword on components: psychological, diet and nutrition and physical activity.
		Description of main food components, sources of fat, food pyramid and food plate theory. General outlook on physical activity and exercise. Introduction to cognitive and behaviour aspects related to lifestyle alteration. Getting-to-know- you session lasting half a day to help participants get acquainted with each other.
**2**	Group session	**Calorie measurement and healthy food options.**
		Education and hands on session for self-monitoring through the measurement of food and calories by participants utilising scales and measurement tools. Reading food labels and using the food plate theory for practice. Sessions with active facilitator participation.
**3**	Group session	**Moving Your Muscles.**
		Information on effective exercise methods (brisk walking). Raise awareness on the safety aspects of physical activity and exercise. Highlight the enhancement of general health through physical activity and exercise. Assess individual's present level of physical activity and exercise. Rise in activity levels to be spread out over a period of time. Set weekly goals for physical activity. Commence self-monitoring for physical activity and exercise. Group activity for aerobic session.
**4**	Group session	**Negative thoughts.**
		Uncover negative thoughts. Increase knowledge on variety of negative thoughts, types of thought disorder and its relationship to behaviour. Suggest ways to offset negative thoughts. Highlight the significance of appreciating oneself for personal accomplishments. Review negative thoughts and set up action plan to counter them.
**5**	Group session	**Relationship between behaviour, environment and healthy lifestyle change.**
		Define social and physical environment and relate its influence on physical activity and eating behaviour. Promote positive response to environmental and social drawbacks. Highlight the need for a healthy diet and physical activity /exercise. Emphasize on acting as role models for group members in order to promote physical activity and positive eating. Appraisal of present behaviour and setting up of action plan to achieve positive behaviour change
**6**	Group session	**Consolidating Exercise Programme**
		Initiate aerobic fitness sessions and define frequency, intensity, time and type of activity (F.I.T.T). Educate participants on the engagement of self-monitoring to set up an effective exercise schedule through the calculation of heart rate and rate of perceived exertion (RPE).
		Discuss schedule and include a variety of activities and exercises to develop flexibility, strength, cardiovascular health and endurance.
**7**	Seminar	**Consolidate dietary, fitness, thought and behaviour alteration.**
		Report on the progress of participants. Reassess weight loss goals, diet and physical activity based on the most recent measurements. Behaviour: Initiate stress management procedure (abdominal breathing) and problem solving techniques (I.D.E.A.L approach) to deal with issues that crop up due to alterations in diet and daily activities. Dialogue on problems encountered by participants. Encourage healthy eating through the promotion of high volume and low calorie foods.
		Dietary: Educate on the choice of healthy food when eating out. Explore by group members of ways to be more active on busy work days.
		Fitness: Suggest activities for work related sitting time. Stress on the need for intervals between extended periods of sitting especially at work. Discuss options on exercise location during unfavourable weather conditions. Teach ways to improve cardiovascular health through resistance and strength exercises. Set up a physical activity schedule for the following month.
**8**	Group session	**Challenges in Lifestyle change.**
		Discourse among group members on problems encountered during attempts to change their lifestyles focussing on eating habits and physical activities. Exchange of information and problem-solving suggestions among participants. Re-assess obstacles faced and define a strategy to overcome these barriers to change.
**9**	Group session	**Maintaining motivation.**
		Concentrate on means to be constantly motivated for lifestyle change. Stress on awareness of internal and external sources of motivation. Emphasise on appreciating minor accomplishments related to lifestyle change. Initiate discussion among group members on strategy to maintain motivation.
**10**	Seminar	**Experience sharing**.
		Disclosure of participants’ accomplishments during the course of the programme (6 months). Initiate panel discussion involving team and group members on the achievements of participants during the programme. Encourage participants to share their six-month experience on the road to better health through weight reduction. Participants were also encourage to continue healthy lifestyle.

A starter kit containing handouts, examples of food energy intake and activity-based energy expenditure in the form of wheel chart was provided to participants during the first session. Handouts were also given to participants after subsequent sessions. Self-monitoring activities included weighing-in, submission of diet records and physical activity log was also conducted for each session. Feedback was provided to motivate the participants on each session.

### Comparison

Participants in the comparison group underwent individual counseling with a dietician once every 12 weeks. Each dietary counseling session lasted for an hour. Recommendations on reduced calorie intake for weight loss, education on food pyramid, self-monitoring of calorie intake, lowering high-fat food and increase of fruits and vegetable intake were given. Participants were advised to increase their physical activity levels and exercise at least three times a week, but no practical session provided.

### Measurements

#### Sociodemographic and lifestyle history

Sociodemographic characteristics included age, gender, ethnicity, education, household income and marital status; family history of diseases such as hypertension, diabetes and hyperlipidemia; and socially related lifestyle behavior such as smoking and alcohol consumption assessed using self-administered questionnaire. Participants were required to fill a three-day dietary record using a log book. They were taught to estimate food intake with standard day-to-day food utensils. Food items according to meals were entered. A food list with energy (kcal) values was given to assist participants’ in self-monitoring for healthy food choices. Physical activity was measured using the International Physical Activity Questionnaire short form (IPAQ-SF). The IPAQ-SF comprised of seven items reported in terms of METS-min per week which can be summarised into three main categories (low, moderate and high). It is a valid measurement of physical activity and has moderate to high reliability [33].

#### Anthropometry, biochemical and clinical measure

Weight was measured using SECA digital scale (Model HD 309) and height was measured using SECA body meter (Model 208) to the precision of 0.1 kg and 0.1 cm, respectively. Participants wore light clothing, and stood on scale without shoes with leg apart within the scale footprint. Waist and hip circumference were measured using non elastic SECA measuring tape to the nearest 0.1 cm following the WHO guideline [34]. Digital OMRON Model HEM 907 was used to measure blood pressure based on the Malaysian Clinical Practice Guidelines for management of Hypertension [35]. Fasting blood samples (after 8 hours) collected by trained staff and sent within four hours of collection in an icebox to the laboratory. Measurements were conducted by trained research assistants at baseline, 12, 24 and 36 weeks. Height measured only once at baseline. BMI was calculated as weight in kilograms divided by height in meters squared.

#### Psychological measures and quality of life

Psychological measures and quality of life were measured using self-administered questionnaires at baseline, 24 and 36 weeks. Weight efficacy lifestyle (WEL) questionnaire was used to measure eating self-efficacy in weight management with 20 items in five subscales. The subscales include food control in negative emotions, availability of food, social pressure, physical discomfort and physical activity with internal consistency ranged from 0.7 to 0.9 [36]. The Malay version of WEL questionnaire had internal consistency (Cronbach’s alpha) of 0.47 to 0.86 [37].

Social support was measured using the Multidimensional Perceived Social Support (MDPSS) which comprised of 12 items and three subscales. Internal reliability achieved was 0.82 for perceived social support from family, 0.88 for friends and 0.94 for significant others [38]. The Malay version of MDPSS has internal consistency of 0.89 with reliability coefficients for family, friends and significant others subscales ranging from 0.67 to 0.83 [39].

Automatic Thought Questionnaire (ATQ) used to assess negative thoughts [40]. Due to poor construct, the Malay version was reduced to 17 items and two subscales from 30 items [41]. Each subscale i.e. negative self concept (NSE) and personal maladjustment (PMA) had internal consistency (Cronbach alpha) of 0.91 and 0.83 respectively. Method of scoring of the Malay ATQ remained the same as the original 30 item with a cut-off score of 30 for depression.

Quality of life was measured using the WHO Quality of Life short form questionnaire (WHOQoL-BREF), a shorter version of WHO Quality of Life (WHOQoL) questionnaire with the objective to measure a global perspective of quality of life across cultures [42]. The WHOQoL-BREF comprises of 26 questions in four domains i.e. physical activity, psychological, social support and environment. Validation of the translated Malay version revealed high reliability with internal consistency of 0.64–0.80, and test—retest-reliability of 0.49–0.88 [43,44].

### Sample size

Sample size was calculated using OpenEpi software. Based on Renjilian et al.’s findings (17), 49% of group therapy participants achieved ten percent weight loss compared to 29% in individual counseling. A total of 286 participants was required after setting the study power at 80%, level of significance at p<0.05 and attrition rate of 25%. Due to low response rate, high number of excluded respondents and time constraint for further recruitment, the sample size was not achieved. Power calculation revealed the sample size of 194 achieved 88.8% power for targeted weight loss.

## Statistical analysis

IBM SPSS Statistics, Version 20 for Windows (SPSS Inc, Chicago IL) used for data analysis. The dietary data was cross-checked and entered by a trained dietician into Nutritionist Pro where the Malaysian nutrient database was used. Between group mean differences at baseline for continuous variables were analyzed using t-tests and χ^2^ tests for categorical data. Mean differences between groups over time used analysis of variance for repeated measures. All analysis was carried out using the intention to treat principles. Missing data was imputed using the last observation carried forward. Continuous data at baseline was described as means± standard deviation. Repeated measure data for within group measures was described as mean ±standard error. Confidence interval (CI) was reported where appropriate. Statistical significance was preset at 0.05. Relative risk reported for proportion achieved for weight loss, while Cohen’s d used for the within and between group repeated measure. Adjustment conducted for measures where the baseline values showed significant between-group differences to control for bias i.e. carbohydrate percentage and multidimensional perceived of social support.

## Results

### Baseline characteristics

A total of 194 participants recruited and randomised. The participants’ mean age (standard deviation) was 40.5 (9.3) years. Majority of participants were women (72.7%) and non-academician (92.3%). More than half (64%) had at least secondary education. A total of 81 (83.5%) GSLiM participants and 80 (82.5%) of dietary counseling participants completed the study at 24 weeks (Fig 1). Participants from the GSLiM group had higher total MDPSS score, family support and friend subscales score as well as carbohydrate percentage intake. No other significant difference between groups was observed at baseline (Tables 4 and 5).

**Table 4:** Baseline Measures.

	GSLiM, mean (sd), n = 97	Diet counselling, mean (sd), n = 97	p value
Age, years	39.7 (9.2)	40.4 (9.5)	0.617
Gender			0.629
Female, n (%)	69 (71.1)	72 (74.2)	
Male, n (%)	28 (28.9)	25 (25.8)	
Ethnicity			0.296
Malay, n (%)	87 (89.7)	91 (93.8)	
Non Malays, n (%)	10 (10.3)	6 (6.2)	
Education, tertiary, n (%)	39 (40.2)	29 (29.9)	0.132
Household income (RM)	2680.34 (1511)	2504.19 (1641.20)	0.438
Married, n. (%)	76 (78.4)	74 (76.3)	0.732
Occupation, academician, n (%)	9 (9.3)	6 (6.2)	0.420
Family history of:			
Hypertensive, n (%)	11 (11.3)	13 (13.4)	0.663
Diabetes, n (%)	3 (3.1)	2 (2.1)	0.683
Hyperlipidaemia, n (%)	8 (8.2)	13 (13.4)	0.248
Behaviour:			
Non-alcohol intake,	96 (99)	97 (100)	0.316
Smoker, n (%)	9 (9.3)	5 (5.2)	0.267
Height, m	1.59 (0.08)	1.58 (0.09)	0.863
Weight, kg	82.3 (16.3)	82.0 (13.5)	0.911
BMI, kg/m^2^	32.4 (4.8)	32.4 (3.8)	0.980
Waist circumference, cm	95.1 (11.2)	94.9 (9.5)	0.872
Hip circumference, cm	112.2 (10.4)	111.1 (8.2)	0.429
Systolic blood pressure, (mmHg)	128.6 (16.1)	129.1 (14.4)	0.712
Diastolic blood pressure, (mmHg)	83.0 (12.2)	83.4 (10.7)	0.774
Triglyceride, (mmol/l)	1.41 (0.62)	1.56 (0.82)	0.182
Total Cholesterol, (mmol/l)	5.2 (0.9)	5.3 (1.1)	0.913
HDL Cholesterol, (mmol/l)	1.23 (0.29)	1.25 (0.31)	0.592
LDL Cholesterol, (mmol/l)	3.45 (0.82)	3.35 (0.85)	0.426
IPAQ SF total METmin^-1^ per week,	1071 (3.24)	1318.3 (3.06)	0.238
Weight efficacy lifestyle	123.0 (25.6)	121.6 (23.4)	0.697
Negative emotions	25.6 (6.5)	25.1 (6.3)	0.560
Availability	21.6 (6.6)	21.4 (6.1)	0.901
Social Pressure	22.3 (6.1)	22.1 (5.7)	0.771
Physical Discomfort	27.7 (5.3)	26.9 (5.6)	0.315
Positive activities	25.8 (5.7)	26.1 (5.2)	0.693
Multidimensional social support ^a^	66.3 (9.9)	62.8(8.7)	0.010^b^
Family Support	22.8 (3.7)	21.7 (3.3)	0.029^c^
Friend Support	20.5 (3.9)	19.3 (3.4)	0.021^c^
Significant Others	22.9 (4.1)	21.8 (4.5)	0.061
Total automatic thoughts questionnaire score	26.5 (7.4)	26.7 (8.0)	0.867
Negative self-concept	16.1 (4.6)	16.2 (4.6)	0.853
Personal mal-adjustment	9.1 (2.8)	9.2 (3.5)	0.822
WHOQoL BREF			
Physical Health	12.9 (1.6)	13.1(1.4)	0.373
Psychological	13.1 (1.7)	13.1 (1.7)	0.942
Social Relationship	14.8 (2.7)	14.6 (2.2)	0.681
Environment	14.1 (1.8)	14.0 (1.9)	0.722

^a^ Significant p<0.001.

^b^ Significant p<0.01.

^c^ Significant p<0.05.

**Table 5:** Baseline Dietary Intake.

	GSLiM, mean (sd) N = 65	Diet counselling, mean (sd), n = 56	p value
Dietary energy intake, mean kcal/day	1421.2 (332.3)	1351.3 (369.8)	0.276
Protein (%)	15.8 (2.3)	15.0 (2.8)	0.109
Carbohydrate (%)	53.3 (6.5)	55.8 (6.1)	0.034^a^
Fat (%)	30.8 (5.4)	29.0 (5.1)	0.064
Cholesterol (mg)	168.0 (88.6)	162.3 (107.2)	0.748
Saturated Fat (mg)	8.1 (3.9)	9.0 (4.5)	0.201
Mono saturated fat (mg)	7.3 (3.6)	7.2 (3.0)	0.772
Meals frequency	3.4 (0.6)	3.6 (0.7)	0.068

^a^ Significant p<0.05.

### Between group differences

At week 24, nineteen participants (19.6%) achieved 6% targeted weight loss in the intervention compared to 4 (4.1%) in the comparison group, Risk Ratio (RR) of 4.75 (95% CI: 1.68, 13.45). Out of 23 participants achieved weight loss, 10 (57.5%) intervention participants and 3 (75%) from the comparison group retained the targeted weight loss (6%) at 36^th^ week. However, when measured in terms of weight in kg, no significant difference observed for the mean difference in weight loss between groups (-0.82 ± 2.12 kg), Cohen’s d 0.001.

Table 6 summarises obesity and cardiovascular measures from baseline to 12^th^ week, 13^th^ to 24^th^ week, baseline to 24^th^ week and from 25^th^ week to 36^th^ week. No other significant mean difference between groups observed at any of the time intervals. Physical activity, psychological and quality of life scores measure from baseline to 24^th^ week and from 25^th^ week to 36th week are shown in Table 7.

**Table 6:** Obesity and Cardiovascular Outcomes Between and Within Groups over time.

	Within Group		Between groups		
	GSLiM mean change (s.e.) *N* = 97	Comparison mean change (s.e.) *N* = 97	Mean difference (95% CI)	Cohen’s d	p value
**0 to 12 weeks**	**(intensive phase)**				
Weight (kg)	***-1*.*52 (0*.*36)***^a^	-0.09(0.19)	- 0.47 (4.69, 3.74)	0.000	0.825
BMI (kg/m^2^)	***- 0*.*60 (0*.*14)***^a^	-0.31(0.08)	- 0.30 (-1.52,0.92)	0.001	0.627
Waist circumference (cm)	***- 1*.*67 (0*.*48)***^a^	0.54 (0.39)	- 0.87 (-3.79, 2.06)	0.002	0.561
Hip circumference (cm)	-0.74(0.39)	-0.16 (0.27)	0.77 (-1.81, 3.35)	0.002	0.557
SBP (mmHg)	0.24 (1.19)	-2.26 (1.01)	0.43 (-3.59, 4.46)	0.000	0.832
DBP (mmHg)	-0.25(0.96)	-1.38 (0.72)	0.09 (-2.91, 3.09)	0.000	0.951
Fasting serum triglyceride (mmHg)	0.02 (0.05)	0.009 (0.047)	-0.14 (-0.33, 0.07)	0.009	0.184
Fasting serum cholesterol (mmHg)	-0.04 (0.06)	-0.04 (0.05)	-0.01 (-0.26, 0.23)	0.000	0.913
HDL (mmHg)	-0.031 (0.017)	0.018 (0.016)	-0.05 (-0.13, 0.04)	0.007	0.256
LDL (mmHg)	-0.058 (0.055)	-0.049 (0.057)	0.09 (-0.12, 0.31)	0.004	0.401
Fasting blood glucose (mmHg)	-0.003 (0.086)	0.121 (0.087)	-0.11 (-0.39, 0.17)	0.003	0.442
**13 to 24 weeks**	**(fade frequency)**				
Weight (kg)	***-0*.*92 (0*.*26)***^b^	***-0*.*61(0*.*22)***^b^	-1.35 (-5.54, 2.85)	0.002	0.527
BMI (kg/m^2^)	***-0*.*36 (0*.*10)***^b^	-0.24 (0.08)	-0.64 (-1.87, 0.58)	0.006	0.300
Waist circumference (cm)	0.16 (0.44)	0.18 (0.37)	-1.79 (-4.73, 1.14)	0.008	0.229
Hip circumference (cm)	-0.44 (0.29)	-0.27 (0.23)	0.39 (-2.21, 3.00)	0.000	0.763
SBP (mmHg)	0.17 (1.08)	0.27 (0.90)	1.63 (-2.37, 5.65)	0.003	0.424
DBP (mmHg)	-1.07 (0.82)	1.27 (0.85)	-0.51 (-3.51, 2.49)	0.001	0.737
Fasting serum triglyceride (mmHg)	-0.05 (0.05)	-0.047 (0.042)	0.01 (-0.24, 0.37)	0.007	0.911
Fasting serum cholesterol (mmHg)	0.08 (0.05)	-0.02 (0.04)	-0.13 (-0.35, 0.09)	0.000	0.239
HDL (mmHg)	0.038 (0.018)	0(0.013)	-0.05 (-0.14, 0.03)	0.008	0.215
LDL (mmHg)	0.033(0.049)	0.039(0.041)	0.08 (-0.4, 0.302)	0.003	0.453
Fasting blood glucose (mmHg)	-0.088(0.046)	-0.070(0.117)	-0.21 (-0.54, 0.12)	0.008	0.207
**0 to 24 weeks**					
Weight (kg)	***-2*.*42 (0*.*49)***^a^	***-0*.*69 (0*.*27)***^c^	-0.82 (-5.00, 3.37)	0.001	0.701
BMI (kg/m^2^)	***-0*.*96 (0*.*19)***^a^	***-0*.*27 (0*.*11)***^b^	-0.44 (-1.65, 0.78)	0.003	0.480
Waist circumference (cm)	***-1*.*51(0*.*52)***^a^	-0.31 (0.12)	-1.12 (-4.00, 1.77)	0.003	0.445
Hip circumference (cm)	***-1*.*18 (0*.*45)***^c^	-0.43 (0.34)	0.62 (-1.96, 3.19)	0.000	0.850
SBP (mmHg)	0.41 (1.23)	-1.99 (1.14)	0.82 (-3.09, 4.72)	0.000	0.680
DBP (mmHg)	-1.32 (0.91)	-0.11 (0.99)	-0.49 (-3.4.1, 2.41)	0.003	0.736
Fasting serum triglyceride (mmHg)	-0.03 (0.06)	-0.04 (0.05)	-0.13 (-0.34, 0.07)	0.009	0.199
Fasting serum cholesterol (mmHg)	0.04 (0.07)	-0.02 (0.06)	0.005(-0.24, 0.25)	0.000	0.970
HDL (mmHg)	0.01 (0.02)	0.02 (0.02)	-0.04 (-0.13, 0.04)	0.002	0.302
LDL (mmHg)	-0.03 (0.07)	-0.01 (0.06)	0.09 (-0.13, 0.30)	0.003	0.418
Fasting blood glucose (mmHg)	-0.09 (0.08)	0.11 (0.09)	-0.16 (-0.45, 0.13)	0.005	0.299
**25–36 weeks (follow-up)**					
Weight (kg)	0.19 (0.35)	-0.11 (0.16)	-1.42 (-5.65, 2.82)	0.002	0.510
BMI (kg/m^2^)	0.02(0.07)	-0.04 (0.64)	-0.67 (-1.92, 0.57)	0.006	0.286
Waist circumference (cm)	0.19 (0.35)	-0.19 (0.37)	-1.44 (-4.39, 1.52)	0.005	0.338
Hip circumference (cm)	-0.45 (0.31)	-0.33 (0.21)	0.26 (-2.39, 2.91)	0.001	0.636
SBP (mmHg)	-1.70 (1.27)	0.46 (0.90)	0.51 (-3.57, 4.48)	0.000	0.802
DBP (mmHg)	-0.72(0.97)	-1.38(0.78)	-1.03 (-4.08, 2.03)	0.002	0.509
Fasting serum triglyceride (mmHg)	0.008(0.04)	-0.01(0.03)	0.008 (-0.25, 0.26)	0.007	0.264
Fasting serum cholesterol (mmHg)	0.01(0.06)	0.07(0.04)	-0.13 (-0.36, 0.09)	0.000	0.953
HDL (mmHg)	0.012 (0.015)	0.003 (0.018)	-0.02 (-0.07, 0.11)	0.003	0.489
LDL (mmHg)	-0.033 (0.063)	0.067 (0.044)	0.03 (-0.19, 0.26)	0.001	0.715
Fasting blood glucose (mmHg)	-0.008 (0.048)	-0.065 (0.039)	-0.22 (-0.58, 0.14)	0.006	0.267

CI, confidence interval; GSLiM, Group Support Lifestyle Modification.

^a^ Significant p<0.001.

^b^ Significant p<0.01.

^c^ Significant p<0.05.

All participants with initial weight measurement were included in this intention to treat analysis. From analysis of repeated measures treatment against time with time as the dependent variable. Effect size stated using Cohen’s for between group mean differences.

**Table 7:** Physical activity, Psychological and Quality of Life score between and within Groups over time.

	Within group		Between groups		
	Intervention group mean change (s.e.)(n = 97)	Control group mean change (s.e.)(n = 97)	Mean Difference at intervals (95% CI)	*Cohen d*	*P*
**0 to 24 weeks**					
***Total IPAQ SF Score*, *mets min***^***-1***^ (sd)*	-18.9 (236.5)	90.3(98.2)	-244.3(-790.1, 301.5)	0.004	0.378
Vigorous, mets min-1	-75.5 (149.7)	58.6 (63.9)	-10.5(-296.4, 275.5)	0.000	0.943
Moderate, mets min-1	-6.8 (95.9)	50.9 (59.5)	-238.6(-509.3, 32.2)	0.015	0.084
Walking, mets min-1	63.3 (79.6)	-19.3(43.8)	4.7(-212.0,221.5)	0.000	0.966
***Total WEL score***	***10*.*3 (2*.*46)***^a^	1.78(1.88)	*5*.*62 (-0*.*10*, *11*.*35)*	0.019	0.054
Negative emotions, mean (sd)	***2*.*02 (0*.*57)***^b^	0.07 (0.47)	***1*.*51 (0*.*004*, *3*.*02)***^c^	***0*.*020***	***0*.*049***
Availability, mean (sd)	***2*.*63 (0*.*62)***^a^	0.93 (0.49)	0.96 (-0.58, 2.50)	0.008	0.218
Social Pressure, mean (sd)	***1*.*71 (0*.*60)***^c^	0.52 (0.43)	0.85 (-0.61, 2.29)	0.007	0.253
Physical Discomfort, mean (sd)	***1*.*41 (0*.*48)***^c^	0.36 (0.44)	***1*.*31 (0*.*05*, *2*.*56)***^c^	***0*.*022***	***0*.*041***
Positive activities, mean (sd)	***2*.*52(0*.*54)***^a^	-0.09 (0.40)	0.99 (-0.27, 2.26)	0.012	0.121
^#^ ***Total MDPSS mean score***	1.46 (0.65)	1.09(0.63)	0.70 (-0.20, 1.60)	0.012	0.127
^#^ Friend Support, mean (sd)	***0*.*86(0*.*25)***^b^	0.44 (0.22)	***0*.*41(0*.*08*, *0*.*75)***^a^	***0*.*030***	***0*.*015***
^#^Family Support, mean (sd)	0.10 (0.27)	0.19 (0.25)	0.09 (- 0.25, 0.44)	0.002	0.578
^#^Significant Others, Mean (sd)	0.50 (0.30)	0.45 (0.32)	0.19(-0.21, 0.59)	0.005	0.347
***Total ATQ mean score***	-0.84 (0.68)	-0.40 (0.43)	-0.40 (-2.29, 1.49)	0.001	0.675
Negative self-concept mean score	-0.39 (0.45)	-0.26 (0.25)	-0.19 (-1.35, 0.97)	0.001	0.745
Personal maladjustment mean score	-0.36 (0.26)	-0.19 (0.23)	-0.19(-0.94, 0.57)	0.001	0.630
***WHOQOL BREF***					
Physical health	***2*.*35(0*.*19)***^a^	***1*.*82(0*.*19)*** ^a^	0.07(-0.35, 0.48)	0.001	0.743
Psychological	***1*.*62(0*.*15)*** ^a^	***1*.*52(0*.*16)*** ^a^	0.06(-0.41, 0.52)	0.000	0.784
Social relationship	***0*.*63(0*.*22)***^c^	0.12 (0.17)	0.39(-0.22, 1.01)	0.008	0.210
Environment	***0*.*52(0*.*14)***^b^	***0*.*25(0*.*11)***^b^	0.18(-0.31, 0.67)	0.003	0.471
**25 to 36 weeks (follow up)**					
***Total IPAQ SF Score*, *mets min***^***-1***^ (sd)*	1.1(143.6)	303.1(138.4)	-449.9 (-1015.1,115.3)	0.013	0.118
Vigorous, mets min-1	-63.1 (73.2)	59.4 (79.6)	-138.8 (-437.5, 159.9)	0.004	0.361
Moderate, mets min-1	62.5 (61.5)	102.1 (82.0)	***-287*.*2 (-563*.*2*, *-11*.*2)***^c^	***0*.*021***	***0*.*041***
Walking, mets min-1	1.7 (73.4)	141.6 (63.6)	- 23.9 (-240.8, 192.9)	0.000	0.828
**Total WEL score**	-1.77 (1.45)	0.45 (1.19)	***8*.*76 (3*.*05*, *17*.*47)***^b^	***0*.*045***	***0*.*003***
Negative emotions, mean (sd)	-0.42 (0.39)	0.06 (0.27)	***2*.*24 (0*.*81*, *3*.*67)***^b^	***0*.*305***	***0*.*002***
Availability, mean (sd)	-0.09 (0.35)	0.19 (0.28)	***1*.*68 (0*.*10*, *3*.*25)***^c^	***0*.*022***	***0*.*037***
Social Pressure, mean (sd)	-0.34 (0.34)	0.09 (0.26)	1.23 (-0.29, 2.75)	0.013	0.114
Physical Discomfort, mean (sd)	-0.40 (0.31)	0.31 (0.25)	***1*.*48 (0*.*28*, *2*.*68)***^c^	***0*.*030***	***0*.*016***
Positive activities, mean (sd)	-0.52 (0.36)	-0.79 (0.33)	***2*.*14 (0*.*93*, *3*.*35)***^a^	***0*.*060***	***0*.*001***
^#^***Total MDPSS mean score***	0.35(0.62)	0.46(0.41)	1.46 (-0.26, 3.17)	0.015	0.095
^#^ Friend Support, mean (sd)	-0.10(0.19)	0.11(0.14)	***0*.*80 (0*.*15*, *1*.*45)*** ^c^	***0*.*030***	***0*.*017***
^#^Family Support, mean (sd)	0.27(0.22)	0.16(0.18)	0.29 (-0.35, 0.95)	0.004	0.364
^#^Significant Others, Mean (sd)	0.09(0.31)	0.19(0.18)	0.38 (-0.38, 1.13)	0.005	0.325
**Total ATQ mean score**	-0.19(0.47)	***-0*.*73(0*.*21)***^b^	-0.35 (-2.17, 1.48)	0.001	0.709
Negative self-concept mean score	-0.10(0.30)	***-0*.*47(0*.*37)***^b^	-0.07 (-1.22, 1.07)	0.000	0.901
Personal maladjustment mean score	-0.08(0.19)	-0.19 (0.09)	-0.21 (-0.94, 0.52)	0.002	0.568
**WHOQOL BREF**					
Physical health	***-0*.*92 (0*.*18)***^a^	***-0*.*53(0*.*19)***^c^	0.13(-0.37, 0.64)	0.001	0.609
Psychological	-0.29 (0.19)	-0.12 (0.20)	0.03(-0.49, 0.54)	0.000	0.923
Social relationship	-0.03 (0.17)	0.14 (0.11)	0.56(-0.08, 1.19)	0.015	0.084
Environment	-0.06 (0.11)	0.06 (0.09)	0.21(-0.28, 0.69)	0.004	0.405

CI, confidence interval; GSLiM, Group Support Lifestyle Modification.

^a^ Significant p<0.001.

^b^ Significant p<0.01.

^c^ Significant p<0.05.

All participants with initial weight measurement were included in this intention to treat analysis. From analysis of repeated measures treatment against time with time as the dependent variable. Effect size stated using Cohen’s for between group mean differences.

^#^ MDPSS, adjusted for baseline MDPSS.

* median values instead of mean for physical activity.

The intervention group achieved higher score for negative emotions and physical discomfort of WEL subscales compared to the comparison group from baseline to 24^th^ week. The effects maintained during follow up from 25^th^ to 36^th^ week. Intervention participants achieved higher friend support mean score from baseline to 24^th^ week, even after adjusting for baseline MDPSS score. During the follow up period, except for social pressure, total WEL and other WEL subscales mean score increased significantly compared to the comparison group. However, moderate physical activity in METSmin^-1^ per week reduced significantly in the intervention group compared to the comparison group during 25^th^ to 36^th^ week. The intervention group experienced lower intake in carbohydrate percentage with higher percentage increase in fat intake over baseline to 12^th^ week and 13^th^ to 24^th^ week. No other dietary change was observed (Table 8).

**Table 8:** Dietary Energy intake between and Within Groups over time.

	Within group change		Between groups		
	Intervention mean (s.e)	Control mean (s.e)	Mean difference at intervals	*Cohen d*	*P*
	N = 65	N = 56	(95% CI)		
0–12 weeks					
Dietary intake, kcal	0.41 (38.2)	45.70 (22.76)	47.25 (-79.8, 174.3)	0.005	0.463
Protein (%)	-0.05 (0.29)	-0.19 (0.20)	0.88 (-0.01, 1.77)	0.09	0.053
Carbohydrate (%)#	***-4*.*12 (0*.*82)***^a^	-1.58 (0.67)	***-1*.*78 (-2*.*86*, *-0*.*69)***^b^	***0*.*08***	***0*.*002***
Fat (%)	***3*.*54 (0*.*93)***^b^	1.77 (0.66)	***2*.*69 (0*.*91*, *4*.*47)***^b^	***0*.*07***	***0*.*003***
13–24 weeks					
Dietary intake, kcal	5.06 (12.96)	15.78 (35.53)	19.24 (-113.3, 151.8)	0.001	0.744
Protein (%)	0.08 (0.16)	0.22 (0.24)	0.93 (-0.02, 1.87)	0.03	0.055
Carbohydrate (%)#	-0.19 (0.40)	***-1*.*91 (0*.*51)***^b^	***-2*.*76 (-4*.*87*, *-0*.*64)***^c^	***0*.*05***	***0*.*011***
Fat (%)	0.11 (0.35)	***2*.*02 (0*.*53)***^b^	***2*.*62 (0*.*36*, *4*.*88)***^c^	***0*.*04***	***0*.*023***
25–36 weeks					
Dietary intake, kcal	-6.52 (9.08)	39.15 (23.7)	-8.96 (-139.7, 121.8)	0.00	0.892
Protein (%)	0.12 (0.09)	0.44 (0.19)	0.69 (-0.30, 1.69)	0.02	0.171
Carbohydrate (%)#	-1.18 (0.13)	-1.48 (0.62)	-1.33(-3.61, 0.95)	0.11	0.250
Fat (%)	-0.07 (0.11)	1.05 (0.55)	1.17 (-1.16, 3.51)	0.01	0.322
0–24 weeks					
Dietary intake, kcal	5.46 (38.28)	61.48 (36.17)	36.12 (-86.9, 159.19)	0.003	0.562
Protein (%)	0.13 (0.30)	0.03 (0.20)	0.81 (-0.08, 1.68)	0.001	0.444
Carbohydrate (%)#	***-4*.*31(0*.*88)***^a^	***-3*.*49 (0*.*64)***^a^	***-1*.*84(-3*.*25*, *-0*.*43)***^c^	***0*.*053***	***0*.*011***
Fat (%)	***3*.*65 (0*.*96)***^b^	3.79 (0.82)	1.74 (0.003, 3.46)	0.000	0.050

Confidence interval; GSLiM, Group Support Lifestyle Modification.

^a^ Significant p<0.001.

^b^ Significant p<0.01.

^c^ Significant p<0.05. All participants with initial weight measurement were included in this intention to treat analysis. From analysis of repeated measures treatment against time with time as the dependent variable. Effect size stated using Cohen’s for between group mean differences.

^#^ CHO percentage was adjusted for its baseline value for between group analyses in all interval.

### Within Intervention group

Weight and BMI of the intervention group were reduced during the first 12 weeks (core phase), with smaller reduction in the next 12 weeks (fade frequency). During follow up, there was an insignificant increase in weight and BMI. Reduction in waist circumference occurred in the first 12 weeks but not sustained during fade frequency. An overall significant hip circumference reduction was observed within interval 0 to 24^th^ week, although the reduction was not significant in the core phase or fade frequency.

WEL and its subscales’ scores, friend support subscale score of MDPSS and WHOQOL-BREF domains score increased from 0 to 24^th^ week. However, the improvement was not sustained during follow up, with significant reduction in WHOQOL-BREF physical health score observed during 25^th^ to 36^th^ week. The intervention group experienced reduction in carbohydrate percentage intake mainly during the core phase, resulting in significant reduction from baseline to 24^th^ week. Concurrent fat percentage intake increased during the same time interval. There was no significant change in cardiovascular risks and physical activity measure throughout the study.

### Within comparison group

The comparison group weight decreased only during 13^th^ to 24^th^ week, yet it contributed to the overall significant reduction throughout treatment from 0 to 24^th^ week. During the same 0 to 24^th^ week period, mean BMI decreased with increased in WHOQOL-BREF physical health, psychological and environment domains mean scores.

Significant decreased in carbohydrate and increased in fat percentage intake, occurred from 13^th^ to 24^th^ week, in the comparison group. As per intervention group, the WHOQOL-BREF physical health score decreased in the follow up phase. Apart from these changes, significant reduction was also observed in negative thoughts and the negative self-concept subscale of the ATQ score during follow up period (25th to 36th weeks).

### Adherence to intervention

#### Attendance

Attendance was assessed as part of adherence measure in the intervention group. Of the total six sessions within the first 12 weeks, 33 (17.0%) participants in the intervention group attended more than four sessions, 34 (17.5%) attended three to four sessions while 30 (15.5%) attended two sessions and less. During the fade frequency with four sessions, 20.6 percent participants attended three to four sessions. None of the participants who attended less than three sessions achieved 6% weight loss, while 8.9% of participants attended four to six sessions achieved the targeted weight loss. About half (44.8%) of the participants attended more than seven sessions achieved 6% targeted weight loss (p<0.001). Median attendance was six sessions with inter-quartile range of five sessions. There was positive correlation between attendance and weight loss (r = 0.491, P<0.001).

#### Log book submission

The overall log book submission was unsatisfactory for both groups. Only 46.4% (n = 45) intervention participants submitted their log books at 12^th^ week and 16.5% (n = 16) at 24^th^ week. Only 23.7% (23) comparison group participants submitted their log books at 12 weeks and 19.6% (19) at 24^th^ week. Those who did not submit their log books were of younger age (p = 0.03).

### Adverse effect of the intervention

Two male participants with BMI above 40kg/m^2^ from the intervention group experienced mild soft tissue injury during self-conducted exercise and had undergone treatment. No other serious event reported.

## Discussion

The intervention proved to be more effective in achieving the targeted 6% weight loss, improved self-efficacy in dietary control, and achieved better friend support and quality of life than the comparison (dietary counseling) group. The psychological improvement in the intervention group was sustained post intervention. Although the comparison group also experienced reduction in weight, BMI, improvement in quality of life, and ATQ, there was no improvement in self-efficacy and social support.

A total of 19% of GSLiM participants achieved 6% weight loss. For comparison purpose, we recalculated and found 17.5% of GSLiM participants achieved 7% weight loss. Other GLB based study reported 23 to 37% achievement in targeted weight loss [14]. A most recent GLB based trial on work site reported 29% of participants achieved targeted 7% weight loss [26]. While another study assessed the effectiveness of the coach led GLB programme in primary care setting had 37% participants achieving 7% weight loss at 15 months [45]. Our study had lower proportion of targeted weight loss due to fewer sessions in GSLiM compared to the original GLB-DPP. Earlier study reported for every other lifestyle session attended, weight loss could increase by 0.26% (16).

Our findings concurred with recent review which showed multicomponent behaviour intervention was more effective than single component intervention [46]. The mean weight loss within intervention at 24^th^ week was -2.43 kg (95% CI: -3.75, -1.21, p = 0.001), comparable to another trial which reported mean weight loss of -2.3 kg (95% CI: -2.92 to -1.72, p = 0.001) [47]. Most improvement occured during the first 12 weeks in the intervention group where sessions were more intensive. Earlier findings showed positive association between intensity and magnitude of weight loss [48]. Yet, lower intensity programme with similar effect is still being sought by other researchers [49], as intensive intervention are costly in terms of manpower and materials [50]. Apart from weight loss, significant improvement of the self-efficacy score and its subscales were seen among intervention participants compared to the comparison group, similarly found by other studies [51–53]. The intervention group experienced immediate improvement in social support score at baseline. However, after controlling for the baseline friend support score, significant difference between groups was observed for mean friend support score during the 24 weeks. Similar finding was also reported by a recent cluster randomised trial where healthy eating was found to be supported by friend [54].

Any improved ATQ score would be expected in the intervention group due to the presence of cognitive elements incorporated in the programme, instead the improvement of ATQ score occurred within the comparison group. The use of ATQ to measure negative thought in obesity research remains limited in the presence of other tools used to assess dysfunctional eating behavior associated with obesity [55]. The average cut off value for depressogenic negative thoughts using the 17 item scale of ATQ—Malay in non-clinical population was 30.0 and clinical population was 47.0 [41]. The mean ATQ score for both the intervention and comparison groups was 25.6 and 26.3 respectively, below the cut off value; therefor the significant mean difference found between the intervention and comparison group was most likely due to chance. ATQ was used in our study to measure depressogenic features amongst obese participants and observed further change as treatment progressed. Moderate concurrent validity (r = 0.65) was found between the Malay ATQ and Malay-Beck Depression inventory (BDI), while discriminant analysis achieved 89.3%.

Other than improvement in weight, psychological measures and quality of life, there was no improvement in cardiovascular risks among our participants. Other studies found at least 10% weight loss of baseline weight resulted in changes for the cardiovascular risk parameters [56]. Several other reasons may influence the absence of treatment effect on cardiovascular risks in this study. Our participants were within the younger age group and their clinical indicators were of normal range, therefor further improvement would be less likely. The focus of intervention would also influence the result as other studies targeting clinical indicators with health education in dietary approach managed to result in cardiovascular risks improvement [57,58]. Therefore, weight loss may not necessary resulting improvement of cardiovascular risks factors.

Participants also experienced small reduction in carbohydrate percentage intake and increase in fat percentage intake, as found by previous study [59]. Low carbohydrate diet has been associated with greater weight loss compared to low fat diet [60]. Nevertheless, there has been call that the effectiveness of lifestyle modification not to be overshadowed by the continuous search for the best dietary approach [61] when moderate balanced nutrients intake should be advocated.

Although promising, the results of this study should be interpreted cautiously. Since participants were employees from the university, generalizability of the study remained limited within population of the same setting and may differ with the general population. Self—reported measures in particular self-administered surveys used may result in reporting bias. Negative behavior such as smoking and alcohol consumption may result in underreporting, while physical activity may be over reported. Although the short form IPAQ was advocated to be used in view of time-saving, a recent review [62] found that IPAQ SF tended to overestimate the METs-minutes/ week score. However, the categories of physical activity derived from the long form IPAQ [63] showed similarity with the categories found in our study and another Malaysian based study [3]. Therefore, we foresee the results to be reliable in particular in assessing change over time. Objective measures such as pedometer or accelerometer cited to be more reliable [64] however its utilization is resource consuming and may not be feasible in community setting. Our study only measured dietary self-efficacy, however, recent finding showed that exercise self-efficacy may be a better predictor for weight loss compared to dietary self-efficacy [65]. Considering the continuous development in the cognitive- psychological aspect of obesity intervention, both physical and dietary self-efficacy need to be assessed in future studies.

Self-monitoring is another critical component for weight reduction (39), however the log book submission by both groups was poor although attempts had been made to improve submission. This may further explain the small effect size observed and no improvement in cardiovascular risks although weight loss was achieved within each group. Poor log book submission for physical activity and dietary intake has also been reported in other studies (17). The attendance for group sessions was low compared to other studies [25,45,66]. Yet similar findings to ours was also found in another Malaysian based lifestyle intervention trial [67]. We also found processes such as attendance and log book submission to be correlated with weight loss. Competing time work demand has also been cited as factors affecting attendance for work site intervention [25]. Local culture within the society may influence the attendance towards programme which needs to be explored.

Finally, we did not evaluate environmental support such as availability of healthy food choices and physical activity facilities which is known to influence weight loss.

Nevertheless, this study is the first group based programme derived from the GLB-DPP conducted in Malaysia. It addressed the dynamics of psychological aspect within the lifestyle modification programme based on a theoretical construct which was rarely implemented and monitored in other translational weight loss programme. The randomised controlled design as opposed to single arm trial was used to evaluate its effectiveness compared to an existing dietary programme.

## Conclusion and Recommendations

Our findings showed the group based workplace intervention (GSLiM) programme managed to achieve targeted weight loss, improved self-efficacy and created positive support with lower intensity. The incorporation of group based approach and psychological sessions managed to achieve psychological change needed to achieve weight loss. The GSLiM programme is ready to be used and can be replicated in similar setting with possible enhancement for exercise self-efficacy, attendance, and adherence to self-monitoring. The programme should be extended for longer duration. Further research should explore the predictive value of the psychological factors in assisting participants to achieve their targeted weight loss.

## Supporting Information

S1 File Research Protocol. (PDF)

S2 File Data. (XLS)

S1 Table CONSORT checklist for Randomised Control Trial. (PDF)

## References

[1] Ng M, Fleming T, Robinson M, Thomson B, Graetz N, Margono C, et al (2014) Global, regional, and national prevalence of overweight and obesity in children and adults during 1980–2013: a systematic analysis for the Global Burden of Disease Study 2013. The Lancet 384: 766–781. 10.1016/S0140-6736(14)60460-8 PMC4624264 24880830

[2] Khambalia AZ, Seen LS (2010) Trends in overweight and obese adults in Malaysia (1996–2009): a systematic review. Obes Rev 11: 403–412. 10.1111/j.1467-789X.2010.00728.x 20233309

[3] Ministry of Health M, Committee (2015) National Health Morbidity Report

[4] Proper KI, Singh AS, Van Mechelen W, Chinapaw MJ (2011) Sedentary behaviors and health outcomes among adults: a systematic review of prospective studies. Am J Prev Med 40: 174–182. 10.1016/j.amepre.2010.10.015 21238866

[5] Moore CJ, Cunningham SA (2012) Social position, psychological stress, and obesity: a systematic review. J Acad Nutr Diet 112: 518–526. 10.1016/j.jand.2011.12.001 22709702

[6] Carmienke S, Freitag M, Pischon T, Schlattmann P, Fankhaenel T, Goebel H, et al (2013) General and abdominal obesity parameters and their combination in relation to mortality: a systematic review and meta-regression analysis. Eur J Clin Nutr 67: 573–585. 10.1038/ejcn.2013.61 23511854

[7] Ul‐Haq Z, Mackay DF, Fenwick E, Pell JP (2013) Meta‐analysis of the association between body mass index and health‐related quality of life among adults, assessed by the SF‐36. Obesity 21: E322–E327. 10.1002/oby.20107 23592685

[8] WHO (2008) Noncommunicable diseases country profiles 2014. WHO.

[9] Mustapha FI (11 September 2014) Increasing Burden of Non-Communicable Diseases in Malaysia: Challenges in Resource Allocation. Payor Network Initiatives 2014, Kuala Lumpur.

[10] Wyatt HR (2013) Update on treatment strategies for obesity. The Journal of Clinical Endocrinology & Metabolism 98: 1299–1306. 23443815 10.1210/jc.2012-3115 PMC3615205

[11] Wadden TA, Webb VL, Moran CH, Bailer BA (2012) Lifestyle Modification for Obesity: New Developments in Diet, Physical Activity, and Behavior Therapy. Circulation 125: 1157–1170. 10.1161/CIRCULATIONAHA.111.039453 22392863 PMC3313649

[12] Knowler WC, Barrett-Connor E, Fowler SE, Hamman RF, Lachin JM, Walker EA, et al (2002) Reduction in the incidence of type 2 diabetes with lifestyle intervention or metformin. The New England Journal Of Medicine 346: 393–403. 11832527 10.1056/NEJMoa012512 PMC1370926

[13] Blokstra A, van Dis I, Verschuren WM (2012) Efficacy of multifactorial lifestyle interventions in patients with established cardiovascular diseases and high risk groups. European Journal of Cardiovascular Nursing 11: 97–104. 10.1016/j.ejcnurse.2010.10.005 21130687

[14] Ali MK, Echouffo-Tcheugui J, Williamson DF (2012) How effective were lifestyle interventions in real-world settings that were modeled on the Diabetes Prevention Program? Health Aff (Millwood) 31: 67–75. 22232096 10.1377/hlthaff.2011.1009

[15] Bandura A (1989) Human agency in social cognitive theory. American psychologist 44: 1175 2782727 10.1037/0003-066x.44.9.1175

[16] Bandura A (2001) Social cognitive theory: an agentic perspective. Annu Rev Psychol 52: 1–26. 11148297 10.1146/annurev.psych.52.1.1

[17] Johnson F, Pratt M, Wardle J (2012) Dietary restraint and self-regulation in eating behavior. Int J Obes 36: 665–674. 10.1038/ijo.2011.156 21829162

[18] Schwarzer R (2014) Self-efficacy: Thought control of action: Taylor & Francis.

[19] Leahey TM, Gokee LaRose J, Fava JL, Wing RR (2011) Social influences are associated with BMI and weight loss intentions in young adults. Obesity (Silver Spring) 19: 1157–1162. 21164501 10.1038/oby.2010.301 PMC3079776

[20] Mobbs O, Crépin C, Thiéry C, Golay A, Van der Linden M (2010) Obesity and the four facets of impulsivity. Patient Educ Couns 79: 372–377. 10.1016/j.pec.2010.03.003 20399590

[21] Dalle Grave R, Centis E, Marzocchi R, El Ghoch M, Marchesini G (2013) Major factors for facilitating change in behavioral strategies to reduce obesity. Psychology research and behavior management 6: 101 10.2147/PRBM.S40460 24124398 PMC3794892

[22] Moroshko I, Brennan L, O'Brien P (2011) Predictors of dropout in weight loss interventions: a systematic review of the literature. Obesity Reviews 12: 912–934. 10.1111/j.1467-789X.2011.00915.x 21815990

[23] Verweij LM, Coffeng J, van Mechelen W, Proper KI (2011) Meta-analyses of workplace physical activity and dietary behaviour interventions on weight outcomes. Obesity Reviews 12: 406–429. 10.1111/j.1467-789X.2010.00765.x 20546142

[24] Salinardi TC, Batra P, Roberts SB, Urban LE, Robinson LM, Pittas AG, et al (2013) Lifestyle intervention reduces body weight and improves cardiometabolic risk factors in worksites. Am J Clin Nutr 97: 667–676. 10.3945/ajcn.112.046995 23426035 PMC3607649

[25] Giese KK, Cook PF (2014) Reducing obesity among employees of a manufacturing plant: translating the Diabetes Prevention Program to the workplace. Workplace health & safety 62: 136. 24702680 10.1177/216507991406200402

[26] Kramer MK, Molenaar DM, Arena VC, Venditti EM, Meehan RJ, Miller RG, et al (2015) Improving employee health: evaluation of a worksite lifestyle change program to decrease risk factors for diabetes and cardiovascular disease. J Occup Environ Med 57: 284–291. 10.1097/JOM.0000000000000350 25742535 PMC4351781

[27] Groeneveld IF, Proper KI, van der Beek AJ, Hildebrandt VH, van Mechelen W (2010) Lifestyle-focused interventions at the workplace to reduce the risk of cardiovascular disease-a systematic review. Scandinavian journal of work, environment & health: 202–215. 10.5271/sjweh.2891 20066386

[28] Bilger M, Finkelstein EA, Kruger E, Tate DF, Linnan LA (2013) The effect of weight loss on health, productivity, and medical expenditures among overweight employees. Med Care 51: 471–477. 10.1097/MLR.0b013e318286e437 23632594 PMC3654027

[29] Deurenberg P, Deurenberg‐Yap M, Guricci S (2002) Asians are different from Caucasians and from each other in their body mass index/body fat per cent relationship. Obesity Reviews 3: 141–146. 12164465 10.1046/j.1467-789x.2002.00065.x

[30] Pan CY, So WY, Khalid BA, Mohan V, Thai AC, Zimmet P, et al (2004) Metabolic, immunological and clinical characteristics in newly diagnosed Asian diabetes patients aged 12–40 years. Diabet Med 21: 1007–1013. 15317606 10.1111/j.1464-5491.2004.01287.x

[31] Chen Y, Copeland WK, Vedanthan R, Grant E, Lee JE, Gu D, et al (2013) Association between body mass index and cardiovascular disease mortality in east Asians and south Asians: pooled analysis of prospective data from the Asia Cohort Consortium. BMJ 347: f5446 10.1136/bmj.f5446 24473060 PMC3788174

[32] Kramer MK, Kriska AM, Venditti EM, Miller RG, Brooks MM, Burke LE, et al (2009) Translating the Diabetes Prevention Program: a comprehensive model for prevention training and program delivery. Am J Prev Med 37: 505–511. 10.1016/j.amepre.2009.07.020 19944916

[33] Craig CL, Marshall AL, Sjostrom M, Bauman AE, Booth ML, Ainsworth BE, et al (2003) International Physical Activity Questionnaire: 12-Country Reliability and Validity. Medicine & Science in Sports & Exercise 35: 1381–1395. 12900694 10.1249/01.MSS.0000078924.61453.FB

[34] Consultation WE (2008) Waist circumference and waist-hip ratio. Report of a WHO Expert Consultation Geneva: World Health Organization. pp. 8–11.

[35] Ministry of Health M (2008) Management of Hypertension.

[36] Clark MM, Abrams DB, Niaura RS, Eaton CA, Rossi JS (1991) Self-efficacy in weight management. J Consult Clin Psychol 59: 739–744. 1955608 10.1037//0022-006x.59.5.739

[37] Chang CT (2007) Applicability of the stages of change and Weight Efficacy Lifestyle Questionnaire with natives of Sarawak, Malaysia. Rural Remote Health 7: 864 18076311

[38] Zimet GD, Dahlem NW, Zimet SG, Farley GK (1988) The Multidimensional Scale of Perceived Social Support. Journal of Personality Assessment 52: 30–41. 10.1080/00223891.1990.9674095 2280326

[39] Ng CG, Amer Siddiq AN, Aida SA, Zainal NZ, Koh OH (2010) Validation of the Malay version of the Multidimensional Scale of Perceived Social Support (MSPSS-M) among a group of medical students in Faculty of Medicine, University Malaya. Asian Journal of Psychiatry 3: 3–6. 23051129 10.1016/j.ajp.2009.12.001

[40] Hollon SD, Kendall PC (1980) Cognitive self-statements in depression: Development of an automatic thoughts questionnaire. Cognitive Therapy and Research 4: 383–395.

[41] Oei TPS M F (2008) Exploratory and Confirmatory Factor Validation and Psychometric Properties of the Automatic Thoughts Questionnaire for Malays (ATQ-Malay) in Malaysia. Hong Kong Journal of Psychiatry 18: 92–100.

[42] Huang I-C, Wu AW, Frangakis C Do the SF-36 and WHOQOL-BREF Measure the Same Constructs? Evidence from the Taiwan Population*. Quality of Life Research 15: 15–24. 16411027 10.1007/s11136-005-8486-9

[43] Hasanah CI, Naing L, Rahman AR (2003) World Health Organization Quality of Life Assessment: brief version in Bahasa Malaysia. Med J Malaysia 58: 79–88. 14556329

[44] Hasanah CI, Razali MS (1999) The pilot study of whoqol-100 (malay version). Malays J Med Sci 6: 21–25. 22589685 PMC3329746

[45] Ma J, Yank V, Xiao L, Lavori PW, Wilson SR, Rosas LG, et al (2013) Translating the Diabetes Prevention Program lifestyle intervention for weight loss into primary care: a randomized trial. JAMA Intern Med 173: 113–121. 10.1001/2013.jamainternmed.987 23229846 PMC3856315

[46] Johns DJ, Hartmann-Boyce J, Jebb SA, Aveyard P (2014) Diet or Exercise Interventions vs Combined Behavioral Weight Management Programs: A Systematic Review and Meta-Analysis of Direct Comparisons. J Acad Nutr Diet 114: 1557–1568. 10.1016/j.jand.2014.07.005 25257365 PMC4180002

[47] Dunkley AJ, Bodicoat DH, Greaves CJ, Russell C, Yates T, Davies MJ, et al (2014) Diabetes prevention in the real world: effectiveness of pragmatic lifestyle interventions for the prevention of type 2 diabetes and of the impact of adherence to guideline recommendations: a systematic review and meta-analysis. Diabetes Care 37: 922–933. 10.2337/dc13-2195 24652723

[48] Wadden TA, Neiberg RH, Wing RR, Clark JM, Delahanty LM, Hill JO, et al (2011) Four-Year Weight Losses in the Look AHEAD Study: Factors Associated with Long-Term Success. Obesity (Silver Spring) 19: 1987–1998. 21779086 10.1038/oby.2011.230 PMC3183129

[49] Liu YL, Lu CW, Shi L, Liou YM, Lee LT, Huang KC (2015) Low intensive lifestyle modification in young adults with metabolic syndrome a community-based interventional study in Taiwan. Medicine (Baltimore) 94: e916. 26039125 10.1097/MD.0000000000000916 PMC4616347

[50] Li R, Zhang P, Barker LE, Chowdhury FM, Zhang X (2010) Cost-Effectiveness of Interventions to Prevent and Control Diabetes Mellitus: A Systematic Review. Diabetes Care 33: 1872–1894. 10.2337/dc10-0843 20668156 PMC2909081

[51] Rejeski WJ, Mihalko SL, Ambrosius WT, Bearon LB, McClelland JW (2011) Weight loss and self-regulatory eating efficacy in older adults: the cooperative lifestyle intervention program. The Journals of Gerontology Series B: Psychological Sciences and Social Sciences 66: 279. 10.1093/geronb/gbq104 PMC3078758 21292809

[52] Sallit J, Ciccazzo M, Dixon Z (2009) A cognitive-behavioral weight control program improves eating and smoking behaviors in weight-concerned female smokers. J Am Diet Assoc 109: 1398–1405. 10.1016/j.jada.2009.05.009 19631046

[53] Hays LM, Finch EA, Saha C, Marrero DG, Ackermann RT (2014) Effect of Self-Efficacy on Weight Loss: A Psychosocial Analysis of a Community-Based Adaptation of the Diabetes Prevention Program Lifestyle Intervention. Diabetes Spectrum 27: 270–275. 10.2337/diaspect.27.4.270 25647049 PMC4231937

[54] Wang ML, Pbert L, Lemon SC (2014) Influence of Family, Friend and Coworker Social Support and Social Undermining on Weight Gain Prevention Among Adults. Obesity 22: 1973–1980. 10.1002/oby.20814 24942930 PMC4435839

[55] Katterman SN, Kleinman BM, Hood MM, Nackers LM, Corsica JA (2014) Mindfulness meditation as an intervention for binge eating, emotional eating, and weight loss: a systematic review. Eating behaviors 15: 197–204. 10.1016/j.eatbeh.2014.01.005 24854804

[56] Ellsworth DL, Mamula KA, Blackburn HL, McDyer FA, Jellema GL, van Laar R, et al (2015) Importance of substantial weight loss for altering gene expression during cardiovascular lifestyle modification. Obesity 23: 1312–1319. 10.1002/oby.21079 25960328

[57] Ariffin FD, Ismail AAA, Sean VTP, Yusoff Z, Awang SA, Rani W, et al (2014) Improved insulin sensitivity, central systolic pressure and inflammatory indicators achieved with minor weight reduction in overweight and obese subjects given education on lifestyle modification. Asian Biomedicine 8: 185–194.

[58] Moy F, Sallam AAB, Wong M (2006) The results of a worksite health promotion programme in Kuala Lumpur, Malaysia. Health Promot Int 21: 301–310. 16963785 10.1093/heapro/dal031

[59] Moy FM, Sallam AA, Wong ML (2008) Dietary Modification in a Workplace Health Promotion Programme in Kuala Lumpur, Malaysia. Asia Pacific Jpurnal of Public Health 20: 5. 19533877

[60] Hu T, Yao L, Reynolds K, Niu T, Li S, Whelton PK, et al (2016) Adherence to low-carbohydrate and low-fat diets in relation to weight loss and cardiovascular risk factors. Obesity Science & Practice 2: 24–31. 27114827 10.1002/osp4.23 PMC4840987

[61] Pagoto SL, Appelhans BM (2013) A call for an end to the diet debates. JAMA 310: 687–688. 10.1001/jama.2013.8601 23989081

[62] Lee PH, Macfarlane DJ, Lam TH, Stewart SM (2011) Validity of the international physical activity questionnaire short form (IPAQ-SF): A systematic review. Int J Behav Nutr Phys Act 8: 115–115. 10.1186/1479-5868-8-115 22018588 PMC3214824

[63] Chu AHY, Moy FM (2012) Reliability and Validity of the Malay International Physical Activity Questionnaire (IPAQ-M) Among a Malay Population in Malaysia. Asia-Pacific Journal of Public Health. 10.1177/1010539512444120 22593217

[64] Hallal PC, Andersen LB, Bull FC, Guthold R, Haskell W, Ekelund U Global physical activity levels: surveillance progress, pitfalls, and prospects. The Lancet 380: 247–257. 10.1016/S0140-6736(12)60646-1 22818937

[65] Byrne S, Barry D, Petry NM (2015) Predictors of weight loss success: Exercise vs. dietary self efficacy and treatment attendance (vol 58, pg 695, 2012). Appetite 95: 593–593. 10.1016/j.appet.2012.01.005 PMC3726181 22248709

[66] Seidel MC, Powell RO, Zgibor JC, Siminerio LM, Piatt GA (2008) Translating the Diabetes Prevention Program into an urban medically underserved community: a nonrandomized prospective intervention study. Diabetes Care 31: 684–689. 10.2337/dc07-1869 18252904

[67] Soon HK, Saad HA, Taib MN, Rahman HA, Mun CY (2013) Effects of combined physical activity and dietary intervention on obesity and metabolic parameters in adults with abdominal obesity. Southeast Asian J Trop Med Public Health 44: 295–308. 23691640

