# Supplementary material for: Effectiveness of a Group Support Lifestyle Modification (GSLiM) Programme among Obese Adults in Workplace: A Randomised Controlled Trial
Source: PLoS One. 2016 Aug 18;11(8):e0160343. doi: 10.1371/journal.pone.0160343 (PMC4990415; doi:10.1371/journal.pone.0160343)

Research Protocol for Medical Ethics Committee Submission

Title:

Group Support Lifestyle Modification (G SLiM) Programme versus Dietary Counselling For Obese Adults in University Malaya: A Randomised Controlled Trial

Primary Investigator:

Dr. Siti Noraida binti Jamal

Doctor of Public Health

Year 1 Candidate

Department of Social and Preventive Medicine

Date: 04/03/2011

Contents

Contents 1
Tables 3
1.0 INTRODUCTION 1
2. LITERATURE REVIEW 3
2.1 Overview 3
2.2 Topic Based Literature review 4
2.2.1. Obesity Prevalence in Malaysia 4
2.2.2 Health Effects of Obesity 4
2.2.3 Other Obesity related research in Malaysia 5
2.2.6. Factor Affecting Weight Loss 6
2.2.7 Behaviour Treatment in Obesity Management 7
2.2.8 Role of Psychosocial Support in Chronic Disease Management 8
2.3 The Randomised Control Trials methods (Lifestyle modification based intervention) 8
2.3.1 The Overview 8
2.3.2 Methods 9
2.3.3 Tools 11
2.3.4 Pilot 11
2.3.5 Intervention 11
2.3.6 Control 15
2.3.7 Personnel 15
2.3.8 Results 15
2.3.9 Others 16
3.0 STUDY OBJECTIVES 17
3.1 Research Problem 17
3.2 Hypothesis 17
3.3 Objectives 18
3.3.1 General Objectives 18
3.3.2 Specific Objectives 18
3.3.2.1 Primary 18
3.3. 2.2 Secondary 19
4. METHODOLOGY 20
4.1 Study Design 20
4.2 Study period 20
4.3 Study area / site: 20
4.4. Study population 20
4.5 Sampling frame 21
4.6. Sample size 21
4.6 Recruitment 21
4.7 Screening 22
4.8 Randomisation 24
4.9 Intervention 24
4.10 Control 25
4.11 Quality Control 26
4.12 Rules to stop participation 26
4.13 Study variables, Confounders, Operational Definitions and Scales of Measurements 26
4.14 Scales of Measures 26

4.15 Methods of data collection 27
4.15.2 Continuous 27
4.16. Plan for data collection and processing 29
4.16.1 Data collection plan 29
4.17 Plan for data analysis 30
4.18 Record Keeping 30
4.19 Pre test and validation of study instrument / Pilot 30
5. LIMITATIONS OF STUDY AND MEASURES TO LIMIT THEM 31
5.1 Main Limitations 31
5.1.1 Blinding 31
5.1.2 Contamination/ crossover of groups 31
5.1.3 Weight maintenance and follow up 31
6. ETHICAL CONSIDERATIONS 32
WORKPLAN (GANTT CHART) 33
Appendix 1- Map 37

Tables

Table 1: Benefits of weight loss on health risks in obesity 2
Table 2: Evidence table 3
Table 3: Summary of Intervention and Control of nine RCT 8
Table 4: Study design and study period of the RCTs 9
Table 5: Summary of interventions and control of RCT reviewed. 12
Table 6: Quick Formula for Calculating Calorie Requirement among participants for weight loss *and weight maintenance** 24
Table 7: Summary of Self Administered questionnaire to be used 27
Table 8: Data collection timeframe and validation 29

ABSTRACT

Background: Overweight and obesity prevalent has increased by 154% within a period of 10 years in Malaysia. Lifestyle modification is an integral component in weight management. Social support proves to improve health outcome in chronic diseases, including group support. This study attempts to evaluate the effectiveness of group empowerment lifestyle modification among obese staffs in University Malaya compared to the usual minimal dietetic counselling.

Methods: This parallel, single blinded randomized control trial was conducted in a period of 9 months from 1st May 2011 to 1st January 2012. Three hundred and eighty six (n=386) overweight and obese men and women with BMI>27.5kg/m2 were divided into intervention and control group on 1:1 ratio. A total of 16 sessions will be done within the 24 week period of intervention. One group would consist of 10-12 members and a minimum of 4/5 of the group shall be allowed to proceed with group discussion at any time. The group shall consist of 1 moderator / leader elected from the group member themselves and a facilitator would be made available in case of queries, to discuss the any issues pertaining to weight management.

Measures of outcome: Primary outcome of proportions between groups that achieved targeted weight loss (6% within 6 months), mean weight loss difference between groups were measured at 0, 3 6 and 9 months after the trial begun. Other outcome measures are; within group mean weight loss difference, between and within group mean blood pressure, waist circumference, fasting serum lipids and Fasting Blood Sugar. Intermediate outcome included Perceived Social support and Self Efficacy as well as physical activity and dietary intake were measured. Data will be analysed with intention to treat.

Conclusions: The group support lifestyle modification program may prove to be effective in weight loss and weight maintenance. It should be used by the healthcare provider and community to assist in weight loss management among the overweight and obese.

1.0 INTRODUCTION

Obesity is a global epidemic. It serves as the 5th highest ranked health risk globally (1) for other diseases. The diseases ranges from breathlessness to cardiovascular disease, Type 2 diabetes and cancer (2). It is estimated in 2005, 1.6 billion adult worldwide are overweight with 400 million of them are obese. And that by 2015, there will be 2.3 billion overweight adults in the world and more than 700 million obese(3).

The obesity epidemic affects not only the developed countries, but also the developing regions as result of socioeconomic transition(4).Within the South East Asian region, Malaysia as well as Thailand has the highest obesity prevalence (26.1% for both countries), as compared to Singapore (6%) (5).

The concerns with obesity in particular to Malaysia are: 1) there is a 154% increase in obesity prevalence over a period of 10 years i.e. from 4.4% in 1996 to 14% in 2006(6); 2) the prevalence of associated diseases e.g. Diabetes and hypertension increases within the same period(5); 3) the trend occurs despite availability of National Healthy lifestyle campaign by the Ministry of Health, (MoH) since 1991(7) and 4) as of other developing countries, the effects of the increased prevalence of chronic diseases, already stretched resources faced as part of the double disease burden (8).

A reduction of weight by 10% from the existing weight proves to improve the above clinical parameters as in Table 1. To date, the mainstay of obesity management is lifestyle modification that comprises of three main components i.e. diet, exercise and behaviour therapy(9).

Weight management in Malaysia is parked under the beauty label, thus managed through privatised and commercialised industry. Findings that not only there are misperception of body image among Malaysian (10, 11), there appears to be a dependency towards *over the counter products (OTC)* (11) and commercial weight loss programs with unknown short and long term effects.

Table 1: Benefits of weight loss on health risks in obesity

Health risk	Benefit of 10 kg weight loss in a 100kg subject
Blood pressure	10mmHg reduction of systolic BP
	20mmHg reduction of diastolic BP
Lipids	10% reduction in Total Cholesterol
	15% reduction in LDL Cholestrol
	30% reduction in Triglycerides
	8% increase in HDL-cholestrol
Diabetes	>50% reduction in developing DM
	30-50% reduction in Fasting plasma glucose
	15% reduction in HbA1c
Osteoarthritis	Decrease BMI> 2kg/m associated with more than 50% decreased risk of developing osteoarthritis
Mortality	20-25% reduction in all cause mortality
	30-40% reduction diabetes related death
	40-50% reduction in obesity related cancer death

Source:(12)

Access to managed healthcare is often overshadowed by pre-existing infectious diseases and chronic disease e.g. coronary artery disease, Type 2 diabetes mellitus, and hypertension among others, of which obesity is a risk factor. Therefore, provision of obesity management through lifestyle modification guided by healthcare provider has to be made available away from the already overburdened health clinics is crucial.

Evidence showed that social support affects weight loss (13-16) and improves health behavior change (17, 18). And that group based obesity intervention provides better results than individual approach as well as more cost effective (19, 20).Therefore, a group social support approach in assisting lifestyle modification proves to be highly potential to fill in the gap for the management of obesity in Malaysia.

It is of hope that the outcome of this research would provide evidence based, effective, safe and comprehensive program for the Malaysian obese and overweight based on group social support framework for empowerment to be able to mobilise and find the required support to encourage and sustain weight loss. All, with an ultimate aim (effect) to halt the increasing trend of obesity epidemic in Malaysia.

2. LITERATURE REVIEW

Literatures were obtained from various databases mainly i.e. Science Direct; Cochrane data bases and Google Scholar.

2.1 Overview

The literature review in this paper attempts to cover the focus of the study at of each section. Keywords involved concentrates mainly on: social support, groups, obesity and overweight, health effects, and Body Mass index (BMI), group versus individuals, management and /or intervention of obesity and/or overweight, nutrition and /or dietary requirements and randomised control trial. Articles are critically appraised and included for review if 1) It fits the topic of interest, 2) provides strength for the focus topic and 3) includes Randomised Controlled Trial (RCT), Cohort, Cross sectional, descriptive, and experts report. Each paper are assessed in reference to Evidence Table 2 and evaluated on basis of quality (US Preventive Task Force). The modified Van Tulder 1997 classification used for Randomised Control Trial, RCT and modified STROBE for other types of studies including Descriptive studies, case series, cross sectional, case control and cohort studies, whereas for review the PRISM is used.

Table 2: Evidence table

**Grades of evidence based on purported quality of study design**	
Categories of evidence	
I	Evidence obtained from at least one properly from randomised control trial
II	Evidence obtained from well designed controlled trials without randomisation
II-2	Evidence obtained from well designed cohort or case control analytical studies from more than one centre or research group
II-3	Evidence from multiple time series studies with or without intervention. Dramatic results in uncontrolled experiments (such as the results of the introduction penicillin treatment in the 1940s) could be regarded as this type of evidence
III	Opinions of respected authorities based on clinical experience; descriptive studies and case reports; or reports of expert committees

IV	Evidence from expert committee reports or opinions or clinical experience of respected authorities, or both

Source from R.J Coker (21). Reviews are not classified.*

2.2 Topic Based Literature review

The following are the literature discussed based on the topic searched.

2.2.1. Obesity Prevalence in Malaysia

The only systematic review on obesity prevalence in Malaysia from 1990 – 2009 was done by Khambalia and Seen (2010)(6). It summarises 28 Malaysian based research that has overweight / obesity prevalence as an outcome. Results indicates there was a small rise in overweight in 1996, 2004 and 2006(20.7%, 26.7% and 29.1%) and a dramatic increase in obesity for the same period inclusive of 2003 (5.5%, 12.2%, 12.3% and 14.0%). The results also showed that there is a higher preponderance towards female and it is highest among adults 40- 49 years old, with ethnicity involvement highest among Indians, followed by Malays, Chinese and Aboriginals.

2.2.2 Health Effects of Obesity

Literatures reviewed are from primary research and reviews, associate obesity with various ill heaths without contradictory.

2.2.2.1 Malaysia

A study by S. Rampal (22) in determining association between different ethnic groups and the on Prevalence, Awareness, and Control of Diabetes in >30 years old Malaysians reveals that overweight and obesity are the 3rd factor associated with diabetes prevalence after family history and increase of age. The adjusted odds ratio (OR) showed that for those overweight (BMI 25-30kg/m2) have an adjusted OR of 1.37 (95% CI 1.13, 1.66) and those who were obese (>30kg/m2) had an adjusted OR of 1.85% (95% CI 1.46, 2.35), both $p<0.01$.

In relation to hypertension, L. Rampal (23) did similar association with a well represented sample of 16, 440 subjects using a cut off point age 15 and more. Findings reveal that the prevalence of hypertensions correlates with increase of

BMI second after increasing of age, surpasses that of family history. The adjusted odds ratio (OR) showed that for those overweight (BMI 25-30kg/m2) have an adjusted OR of 4.5 (95% CI 3.6, 5.6) and those who were obese (>30kg/m2) had an adjusted OR of 8.1% (95% CI 6.3, 10.3). However, there was no statistical significance given. Prevalence of smoker in obesity was not quoted in NHMS III but showed in Rampal et al (24). i.e. lower risk of obesity in smoker OR=1.52 (95% CI:1.27, 1.81) as compared to non smoker.

Sherina et al (25), also found significant association between marital status and obesity among women OR=2.63 (95% CI 1.90-3.65), as well as found education level OR is 0.55 (95% CI=0.35-0.87).

2.2.3 Other Obesity related research in Malaysia

2.2.3.1Perception of Body Weight

Findings from Fatima et al (26) in assessing perception of bodyweight status in government department in Kuala Lumpur, showed that although there is a high prevalence in obese (38.1%), 21.7% actually perceived themselves as normal weight of which 32.3% are men and 12.3% are women. And third person's perception on the subjects' bodyweight status is significantly associated with the subjects' bodyweight status (P<0.05). However, this study has its limitations in terms of its quality with specifics to its methodology where the sample size was only 500 with a response rate of 77% from an intended larger sample sized that was not discussed. And also that it should be interpreted within the context that it is done within the government sector involving only the Malay ethnics.

2.2.3.2Intention to Lose Weight

While Chang et al (10), using the Stages of Change (SOC) and Weight Efficacy Lifestyle (WEL) among 3 ethnics (Iban, Bidayuh and Malay) in Sarawak village identifies a large proportion of the overweight or obese adults were not intending to lose weight. And those who actually do wanted to lose weight was found by Kong et al (11) in a purposive sampling sample from selected supermarkets in Kuala Lumpur that 60.6% respondents of adults obtained their weight-loss products from the pharmacies. Kong's study however, is not representative of Malaysians.

2.2.3.3 Intervention

Intervention studies up to date are still limited in Malaysia. Moy et al (27) did a quasi experimental study among guards in University Malaya and compared them to the University Malaya Medical Centre Guards upon addressing intensive lifestyle modification in terms of dietary intake and physical activity in the intervention group (N=102) and minimal education through email and group counselling in the control (N= 84). The results showed, statistically significant reduction in mean total cholesterol levels with intervention effect of -0.38 (95% CI=-0.63,-014) mmol/l.

Community based research was done and abstract of articles presented in conferences findings:

- Triple arm study with intervention testing resistant exercise and dumb bell exercise to promote weight loss. (Promoting Weight Loss among Obese Participant Community-based Behavioral Intervention Program in Kelantan) by Wan Suriati (28)
- Competition based lifestyle intervention programme. The outcomes of the program were assessed for its weight loss efficacy, attitude change and quality of life. (Community Lifestyle Intervention Program on Obesity control, Norawai et al Malaysian Family Physician 2009; Volume 4, Supplement 1)(29)

2.2.6. Factor Affecting Weight Loss

An evaluation of the Look AHEAD (Action for Health in Diabetes) Study: Factors associated with success by Wadden et al (40) reveals, after the 1st year of intervention, there was a statistically significant achievement in targeted weight loss of >5.5% for the intensive lifestyle intervention (ILI) group of 8.6% of initial weight as compared to Diabetic Support and Education (DSE) 0.7%; ($p<0.0001$). The predictors identified are physical activity being the strongest, followed by treatment attendance and consumption of meal replacements.

Diet is also one of the identifiable modifiable risk factor in weight loss. Sartorelli et al (41) evaluated the increase of 100 g/d of vegetables and fruits

represented a body weight loss of 500 and 300 g after 6 months, respectively (P <.05). However, these findings could be influenced by the physical activity that is present in the intervention and absent in the control i.e. the comparative baseline is not the same.

In a pre determined priori of behavioural and psychosocial predictors in weight loss among overweight and obese women, Teixeira et al (13) found statistically significant that the higher previous diet attempt affects the less successful the outcome (p<0.05) and the higher the self motivation drive the higher the successful weight loss (p=0.02).

Fabricatore et al (14) in assessing predictor to attrition and successful weight loss in 4 intervention groups using Sibutramine alone, Sibutramine and minimal behavior , lifestyle modification only and combined Sibutramine and life modification, found significant between group difference in predictive value towards weight loss only in the Sibutramine and lifestyle modification group OR 3.66 (95% CI 1.44, 10.88),p=0.01 as compared to Sibutramine alone.

2.2.7 Behaviour Treatment in Obesity Management

A high impact RCT by Knowler et al (42) evidently proves that Lifestyle Intervention through health education is more effective than Metformin. The lifestyle intervention achieve 58 % (95 % CI: 48 to 66%) as compared to Metformin 31 % (95% CI, 17 to 43 %) in lowering the incidence of Type 2 Diabetes Mellitus. Lifestyle modification typically provided to groups of 10-20 individuals (during 60-90 minutes sessions) by registered dieticians, behavioural psychologists, exercise specialists or related health professionals. The time period ranges 16 to 26 weeks for an initial period (43). Distinguishing characteristics of behaviour treatment are (44):

i. Clear goal directed
ii. Process oriented either by food, physical activity and thinking habits)
iii. Advocating small approaches instead of large targets

2.2.8 Role of Psychosocial Support in Chronic Disease Management

Social support is effective in motivating a person to lose weight(45). The most common group support is in diseases that are life threatening stigmatised, and causing embarrassment(46). In diabetes, the group consultation offers protection to Diabetes control. Group support shows positive improvement in knowledge and psychosocial functioning as compared to classical approach of family, friends and spouse based on systematic review of controlled intervention in diabetes by Van Dam et al (48). However, his review also reveals that the definition varied among studies making comparison difficult as found by Venderheijen et al (49) in obesity management. Social support has also been identified as one of predictors to weight maintenance as identified by Smiths et al (50).

2.2.9 Psychological Assessment Tool

Weight efficacy lifestyle questionnaire have been used commonly for in assessing self efficacy (51) and have been used in Malaysia settings (10). Whereas the Multidimensional scale perceived social support is commonly use to assess adherence (52).

2.3 The Randomised Control Trials methods (Lifestyle modification based intervention)

The following section will discuss the literature review based on the RCT methods done in relation to group support and/or management of obesity.

2.3.1 The Overview

All the review involved RCTs. Seven of the nine RCTs reviewed did the intervention with specific aim for weight loss in the overweight and obese group. Three other focuses weight loss in those with Metabolic Syndrome (MetS) (55), and weight loss and diabetes (39).

Table 3: Summary of Intervention and Control of nine RCT

RCT	Intervention/s and Control	Objective/s
Wing et al (2006) (53)	Control group versus Group (face to face) intervention versus group (internet based) intervention	Evaluate effectiveness of face to face group delivery as compared to internet in weight maintenance
Ash et al (2006)(54)	Group based cognitive behaviour therapy versus individualised dietetic treatment versus control (information booklet only)	Effect of 8 week group based cognitive behaviour therapy with monthly follow up
Pettman et al (2009)(55)	Education, Practical strategies and group based support versus Control (written copies of national dietary guidelines)	Evaluate effectiveness in minimally prescriptive group based lifestyle intervention in Metabolic Syndrome
McConnon et al (2007)(56)	Internet group versus Control (Printed information at baseline)	Internet based resource and usual (face to face)
La Rose et al (2009)(57)	Behavioural self regulation (BSR) (group) versus Adapted Standard Behavioural treatment (SBT) (group)	Feasibility of recruiting and retaining young adults in brief behavioural weight loss intervention and efficacy of daily self weighing
Renjilian (2001)(19)	Group cognitive behaviour lifestyle modification therapy versus individual cognitive behaviour lifestyle modification therapy	Whether matching obese people to preference in treatment improve weight loss
Waleekhachonlet et al (2007)(20)	Group behaviour therapy versus individual behaviour therapy	Compare group versus individual approach in promoting healthy behaviour
Reickhem et al (2002)(39)	Assessment of Group versus Individual Diabetes Education	Evaluate effectiveness of group delivery as compared to individual diabetic education
Christie et al(58)	Group versus individual phone based obesity treatment for rural women	Effects of behaviour weight loss program delivered in 2 forms

2.3.2 Methods

2.3.2.1 Study design

Table 4: Study design and study period of the RCTs

RCT	Study design	Study period	Sample size, n
Wing et al (2006) (53)	Triple arm; 1 control; 2 interventions	18 months; with assessment at 6, 12 and 18 months	314
Ash et al (2006)(54)	Triple arm; 1 control; 1 intervention; 1 co intervention	6 month intervention and up to 12 month follow up	191

Pettman et al (2009)(55)	Double arm?	16 weeks intervention	153
McConnon et al (2007)(56)	Double arm: 1:1	6 months	180
La Rose et al (2009)(57)	Double arm;	10 weeks intervention, 20 weeks follow up.	40
Renjilian (2001)(19)	Stratified single sided, matched	26 weekly sessions	75
Waleekhachonle et et al (2007)(20)	Parellel, open labelled, single sided trail	Intervention 3 months, follow up at 6 and 12 months.	132
Reickhem et al (2002)(39)	Centre based ; Double arm 1:1 ratio	6 months intervention.	170
Christie et al(58)	RCT double arm	6 months intervention	34

2.3.2.2 Sample size

The mean sample size reviewed in study is n=143.22± 87.31, with minimum of n= 34 to maximum of n=314.

2.3.2.3 Study period

The study period varied but mostly adhered to 6 months intervention period with those taking minimum of 16 weeks. The 10 week intervention by La Rose et al is reasoned due to it being a pilot study for young adults.

2.3.2.4 Recruitment method

All the recruitment method is through volunteerism, mainly using advertisement. This is expected of RCT though it introduces volunteer bias.

2.3.2.5 Setting

The settings of these studies varied. 3 studies based in tertiary hospitals (19, 39, 54). While one(59) from GP practice and the rest are based in community settings.

2.3.2.6 Randomisation

As seen in summary table 13, five (19, 39, 54, 58, 59) of the nine study specified the details in the randomisation as for the other only mentioned randomisation.

2.3.2.7 Blinding

All the study done was an open labelled study, as addresses by McConnon blinding of either participants or researcher are not possible. Renjilian et al has attempted to minimised bias due to non blinding by blinding the outcome assessors. While Christie et al , mentioned masking during randomised.

2.3.2.8 Inclusion and exclusion criteria

The most consistent inclusion criteria used is BMI with exception of three studies (39, 53, 60), followed by ability to walk and non pregnant or breast feeding in women participants. Serious condition was identified as one of the common exclusion criteria medical as well utilisation of physician who verified ability for participants to participate.

2.3.3 Tools

All the trials measured weight as one of the main outcome including of height for BMI measurement. Psychological assessment has been specified by six of the nine RCT (Renjilian, Waleekhachonleat, Christie, Rickheim , La rose, Ash et al).

2.3.4 Pilot

Pilot study was not mentioned by any of the RCTs.

2.3.5 Intervention

Comprehensive lifestyle modification (both diet, physical activity and behaviour) are the basis of all interventions. The variety exists in the approach of delivery either by face to face (groups or individuals), phone and internet.

Self regulation is also an important component emphasised in all the RCT. Food supplement are used as part as of the intervention by Wing et al but proves non significance difference than the face to face approach.

Table 5: Summary of interventions and control of RCT reviewed.

RCT	***Intervention***
Wing et al (2006)	Both intervention arms given a scale and monitoring system based on color codes.<br>Maintained weight (<1.4kg) - green – provided immediate reinforcement and small gifts<br>Weight gain 1.4-2.2kg – yellow zone-use problem solving skills; weight gain of 2.3kg and >- red zone- restart active weight loss efforts.<br>Tool kit provided at start of weight loss.<br>Lifestyle modification (diet and PA) adherences are encouraged.<br>Meeting: weekly 1st month; monthly meeting thereafter. Included are individual weigh in and group session.<br><br>Internet group:<br>Each provided with a laptop and internet line and technical support. There is introductory session on using laptop to assess STOP regain website and message board. Group meeting was done via a chat room.<br><br>Control group:<br>Received a quarterly newsletter with information about diet, exercise, and weight control. No interaction with intervention staff and seen in clinic only for assessments.
Ash et al (2006)	Intervention 1:<br>Fat Boosters Incorporated (FBI), 8 weeks (1 1/2 hour per week for 6 weeks) with 10-12 groups' f/u at wk 8 and mnthly for 6 mths.<br><br>Intervention 2:<br>Individualised dietetic treatment (IDT). Individualised weekly contact with dietician, individualised diet prescription aiming to achieve weight loss of 0.5-1.0kg/week.<br>And an exercise prescription (20-30 min) accumulated exercise most days of the week. Monthly fu visits from week 8 to 6 months. If participants failed fu attempt to call and provide intervention via phone is made.<br><br>Control:<br>Booklet only (BO) as control. Where there is no additional nutritional advice and fu at 3, 6 and 12 months.
Pettman et al (2009)	Intervention:<br>Shape up for life. Comprised of information and physical activity session delivered by study coordinator (with health and nutrition background). No initial dietary or lifestyle consultations were conducted and NO individual counselling. Information given on benefit of lifestyle choice for maintaining and improving health, individual was left to implement behavioural changes best fit their style. If participants unable to attend sessions attempts made to provide relevant face to face session via email./ phone.<br>Dietary education on providing information aimed to improve the quality of diet based on Australian energy guidelines rather than restricting calorie. Supermarket tour to encourage and read labels. Practical cooking sessions to show methods on how to prepare healthy meals.<br>Self management skills (action plan, problem solving)

	Free food samples as example of healthy food choice not as meal replacements. Information was also given on PA, benefits of regular exercise for maintaining and promoting health and participants are encouraged to try and achieve national PA. Cardio gym was made freely available<br><br>Control: Written copies of Australian national healthy eating and Australian PA guidelines.
McConnon et al (2007)	Combination of dietary, physical activity and behaviour therapy advice. There is an intervention website provides advice, tools and information to support behaviour change, designed to manage their own care. Also provides personalised advice to participants. Motivational statements are also provided. Self reported weight loss, automatic generic webmail are also available. Demonstration of website use was given.<br><br>Control:<br>Usual care to obesity to continue usual approach to weight loss and given small amount of printed information at baseline reflecting that of available in primary care.
La Rose et al (2009)	Both groups have common treatment as follows:<br>Both have the same 10 weekly meeting each at 60 minutes with optional booster session at week 14.<br>Dietary goals:<br>≤91kg 1200 kcal; >91kg 1500kcal. Off 20-30% from fat.<br>Exercise goal:<br>Gradual increase of activity til at least 40 minutes per day for 5 days. Allow accumulation.<br>Behaviour modification: Self monitoring<br><br>Behavioural self regulatory (BSR)<br>Weighing by self using digital memory given (able to store up to 30 days weight information). Participants are to do so each day at the same time (ideally after waking early morning without cloths).Adjustment are to behaviour if fluctuation occurred.<br>Education and self regulation and color zone system<br>Education in principle of self regulation and weight managememnt application as well as diabetes. Scale are used o regulate behaviour. Based on weekly weight, progress are compared to their goals and color zone system used to determine appropriate plan of action. Color zone based on STOP regain modified to weight loss program.<br>Green zone: weight loss> 1kg /week<br>Yellow zone: < 1kg weight loss; use problem solving skills to recert to green zone.<br>Red zone: Gained or remained the same weight ; increase PA and use meal replacement.<br>Maintenance;<br>Maintenance color zone was used.<br><br>Standard behavioural therapy (SBT)<br>Weighed by interventionist before each weekly group meeting. After ended encourage to weigh at home by themselves.<br>Maintenance plan:<br>Interventionist work with participants to formulate goals and action plan for the next month. Participants are encouraged to set specific goals, develop self reinforcement strategies and formulate action plan. Optional booster offered at session week 14.

Renjilian (2001)	Both treatment condition. 26 weekly sessions of standard cognitive bahvioural weight management training (self monitoring, goal setting, stimulus control, etc). Low calorie diet (1200kcal/day, home based exercise 30 mins brisk walking per day 6days per week).<br>Group:<br>Each therapist co led group of 8-12 participants using a behavioural weight management program. Session last 90 minutes. All member s are weight prior session, self monitoring record reviewed (diet, activity, behaviour). Next each group member give brief report of progress since previous sessions. Group leader facilitate group discussion. Final segment of the group session , therapist introduce and explained new eating or exercise related treatment strategy with a written hand out.<br><br>Individual:<br>Each session is 45 minutes. At the outset, participants are weighed and self monitoring records are reviewed including record of daily calorie consumption, exercise and behavioural strategies.<br>Participant report progress since the previous session and therapist provide positive feedback.<br><br>*topics reviewed in both groups are identical.
Waleekhachonle et et al (2007)	All participants were given a goal in achieving 6% weight loss within 6 months. Physical activity was to be maintained. Low calorie diet 1200-1500 kcal/day was recommended. At 2 weeks all together (both groups) received information of reason of healthy eating, strategies of behavioural control for 2hr at community centre setting. Weight control handbook was provided o all for self study. Four sessions (week 4, 6, 8 and 10) conducted at different times to decrease contamination.<br>The difference of these two groups is MODE and TIME of intervention.<br>Intervention 1: Group based.<br>Group meeting.<br>60 minutes per session 3-12 participants. Ratio of provider to participants 1:5.<br><br>Intervention 2: Individual<br>Meet with program provider for 30 minutes per session.
Reickhem et al (2002)	Both individual and group have 4 sessions of diabetes education programme in total of 4-7 hours. The program uses the National standard of Diabetic support education (DSME) for basic education skill. Didactic education limited.<br>Group:<br>Initial visit 3 h. Subsequent 2 week 2h. 3 and 6 mnths 1 h. Classroom set up. Members 4-8 per group.<br><br>Individual<br>Initial visit 2 h. Subsequent 2 week and 3 and 6 months 1h.Individual room
Christie et al	Both groups: Goal to 10% weight loss over 6 months. Weekly treatment sessions 16 weeks followed by biweekly sessions for 4 sessions.(Social cognitive theory by Bandura focused on problem solving, self monitoring. Twice per week participants are required to turn in diet and PA self monitoring logs. Weekly session begun with are view of participants self monitoring logs, followed by questions and problem solving and ended with educational topic on nutrition, PA or cognitive and behaviour strategies. Kcal 1200-1500 kcal/ day.<25% fat. Pre-packaged entree were provided and shakes (shipped directly to their home)gradual increase of PA over the1st 12 weeks 60mins/day, 5 days/week.<br>Group:<br>16 women per group. 60 minutes per group. No multitasking allowed. Calla toll free line for conference.counsellor calls name throughout sessions to ensure participation.

Individual:
Call one to one.25-45 minutes.

2.3.6 Control

Most control has only basic information as such newsletter, information guidelines with no other intervention (53, 54, 59, 60). While the other four studies, (19, 20, 39, 58) compared effectiveness between 2 types of intervention with no control provided.

2.3.7 Personnel

Both Ash and Renjilian et al mentioned on training of interventionist to ensure uniformity of intervention. Pettman's intervention was given by study coordinators (health/nutrition background) with a peer leader. Renjilian used doctoral candidate in clinical psychology and Reickhem utilised RN or diabetic registered nurse specialist. Others did not specifically mention personel used. Uniformity are ensured by training the trainers.

2.3.8 Results

2.3.8.1 Baseline

All except Pettman et al found no significant difference in baseline measurement between group. Pettman found significance findings between groups in the SBP and DBP, however did not elaborate on it.

2.3.8.2 Primary outcome.

With exception of Reickheim et al, Pettman and La Rose, which measures HbA1c,dietary and adherence , respectively, the primary outcome for the other studies is weight loss related.

2.3.8.3 Secondary outcome

Secondary outcome are varied. Clinical parameter e.g BP, HR are mentioned by 3 (20, 39, 60). Other outcome included psychosocial measures with various tool e.g Weight efficacy score.

2.3.9 Others

2.3.9.1 The attrition rate

All has an acceptable range of attrition i.e. <30% with exception to Ash et al of a very high 63% in the control group. The probable reason for this could be that the control has no intervention at al.

2.3.9.2 Non responder

McConnon use strict protocol by post and phone at three attempts, while others did not mentioned handling of adherence by participants.

2.3.9.3 Theoretical construct

Theoretical framework was provided by only 3studies i,.e.Waleekhachonleat, . Reickheim (adult learning model, public health nursing model, health belief model and transtheoretical model) and Christie et al (Social Cognitive Theory).

3.0 STUDY OBJECTIVES

3.1 Research Problem

The main research problem is to evaluate the difference in the effectiveness between a group empowered lifestyle modification and a minimal dietetic counselling that is currently given among the participants of the Wellness clinic.

The research question posed here are:

1. Is there any difference in the proportions between groups that achieved a targeted 6% weight loss within the 6 month intervention period.
2. Is there any difference of mean obesity indicators; (weight (kg) BMI, waist circumference) within and between groups between intervention and self based (control) in lifestyle modification weight based management.
3. Is there any difference of mean difference in clinical outcomes – (blood pressure, fasting blood glucose and cholesterol level) between groups based (intervention) and self based (control) in lifestyle modification weight based management from baseline to end of treatment.
4. Is there any difference in behavior changes (PA & Diet) within and between groups.*
5. Is there any difference in the psychosocial function (perceived social support, self efficacy) between the groups?
6. Is there any a predictive value between perceived social support, self efficacy and weight loss

3.2 Hypothesis

Overall, it is hypothesis based on null hypothesis that there will be no difference between the group empowered lifestyle modification and the usual minimal dietary counselling in the following:

1. There is no statistically significance difference in the proportions between groups that achieved the targeted 6% weight loss within the 6 month intervention period.

2. There is no statistically significance difference in means obesity indicators; (weight (kg) BMI, waist circumference) within and between groups between group based (intervention) and self based (control) in lifestyle modification weight based management.
3. There is no statistically significance difference of mean difference in clinical outcomes – (blood pressure, fasting blood glucose and cholesterol level) between groups based (intervention) and self based (control) in lifestyle modification weight based management from baseline to end of treatment.
4. There is no statistically significance in behavior changes (PA & Diet) within and between groups.*
5. There is no statistically significance in the psychosocial function (perceived social support, self efficacy) between the groups?
6. There is no statistically significance association between perceived social support, self efficacy and weight loss.

3.3 Objectives

3.3.1 General Objectives

Therefore the purpose of this study is to examine the effectiveness of group based social support within a self regulation life style modification obesity management program among participants in the Wellness Program in University Malaya.

3.3.2 Specific Objectives

3.3.2.1 Primary

i. Evaluate the proportions difference of between groups that achieved a targeted 6% weight loss within the 6 month intervention period.

ii. Evaluate mean difference in obesity indicators ;(weight (kg) BMI, waist circumference) within and between groups.*

iii. Evaluate the mean difference in clinical outcomes – (blood pressure, fasting blood glucose and cholesterol level) within and between groups.*

iv. Evaluate difference in behavior changes (PA & Diet) within and between groups.*

*over time

3.3. 2.2 Secondary

i. Evaluate the psychosocial function (perceived social support, self efficacy) with the outcome of groups.

ii. Evaluate the predictive relationship between perceived social support, self efficacy and weight loss.

4. METHODOLOGY

4.1 Study Design

This is a two phased, open labelled, parallel double arm and single blind (outcome assessor blinded), randomized controlled trial to assess the effectiveness of group empowered lifestyle modification program (intervention) as compared to current treatment – minimal dietetic counselling obesity management (control).

The first phase targets weight loss within a 6 month period.

The second phase observes for weight maintenance for 3 months.

4.2 Study period

The study will be conducted from March 2011 to December 2012 i.e. from date of randomisation to completion of follow up.

4.3 Study area / site:

The study will be conducted in among staffs in University Malaya who participate in the Wellness Program in year 2011.

4.4. Study population

The study population consists of adults (>35 years to 65 years) who are obese (27.5kg/m2 < BMI < 35kg/m2) who are staffs of University Malaya between March 2011 to December 2011. (>35kg/m2 are morbidly obese category requires more specific treatment than just lifestyle modification)

Inclusion Criteria:

Age; 18 years to 65 years, Female and males, staffs of University Malaya between March 2011 to December 2011, obese (> 27.5kg), Bahasa Malaysia and/or English Literate, and able to walk briskly at minimum for 10 minutes unassisted

University Malaya is one of the oldest universities in Malaysia. The main campus is located in the Kuala Lumpur on 922 acres (Diagram Appendix 1). The total capacity of the staffs' amounts to 6,861 with a total of 2, 613 academic staff; 590 International Academic staff and 3, 658 total non academic staffs.

4.5 Sampling frame

From 2008 data: out of 1281 participants in wellness clinic (aged 40 and above), 41.8% are overweight (538); 23.1% are obese (296) i.e. **834** potential participants for this study. In 2011, the sampling frame is anticipated to increase slightly as the program is to recruit a staffs from the 35 years and above.

4.6. Sample size

The sample size of this study takes into consideration of the Power: 80%, level of significance: $p < 0.05$ and size of treatment effect:

For the size of treatment effects S Ash et al(54) found the Odds ratio (OR) between dietary self based group as compared to group based (reference) is 0.62 in terms of being sufficiently active, while Renjilian et al (19) found that OR of exposed versus non exposed is 2.33 with intensive individual proportion who achieved targeted weight loss is 29% as compared to 49% in group intervention; $\chi 2=(1,N=75)=2.15$, $p=0.14$.

Anticipating a lower control achievement in this study for the control (only have an existing dietary counselling), and a similar proportional achievement in the intervention (structured group based lifestyle modification), an estimated OR= 2 for the intervention versus control will be use for significance testing.

The anticipated value included in calculation i.e. 25% of those in control and 40% intervention to achieve 6% of weight loss of baseline weight.

In consideration of attrition rate of 25% (19), the total number of required sample in this study is n=386 , with each group ratio of 1:1 is n=193 participants in each arm.

4.6 Recruitment

Recruitment will be done on the opening session for the Wellness Programme in March 2011. It is estimated to continue until the numbers of volunteers are found. Potential participants shall be identified and invitation to participate via letters and/ or phone will be made.

From advertisement to final number of participants in the study is shown in the Recruitment Flow Chart (Appendix 2).

4.7 Screening

Those who are volunteering will be screened to assess eligibility of volunteers. If eligible, a short briefing will be given and consent form issued. Verbal informed consent shall be obtained at this point for the purpose of study as well for purpose of screening. Volunteers shall be briefed of proceeding of being accepted or otherwise by exclusion criteria. Baseline data and blood investigations shall be collected.

Brief history taking and clinical examination shall be conducted by a medical officer/investigator.

Findings such as clinical anemia, hepatosplenomegaly, cardiac murmur and other abnormal findings along with abnormal blood investigations results reviewed will be referred to participants own physicians for further assessment. Letters of fitness to participate by participants own health physician will be required to rule out any undiagnosed serious medical condition.

Staff Nurse/ Medical Assistant:

- Venepuncture for baseline blood investigations as well as screening parameters including Full Blood count, blood urea and serum electrolytes, liver function test, serum cholesterol and fasting blood glucose.
- Measurement of diastolic and systolic blood pressure, height, weight and waist circumference

Exclusion criteria:

- Serious medical risk e.g. unstable cardiac condition, congestive cardiac failure, cancer treatment and / or severe pulmonary disease
- On pharmacotherapy treatment for diabetes, obesity and hyperlipidemia.
- Psychiatry disorders e.g. substance abuse, depression or binge eating disorder
- Weight loss of >5kg in the previous 6 months
- Pregnant or breast feeding
- Planning to get pregnant
- Planning for transfer

Volunteers shall then be informed of acceptance or referral to clinic via telephone call. Those who are accepted or vice versa, will receive a letter of acceptance or decline.

The eligible volunteer shall also be informed of the date of which briefing will be conducted.

Incentive provided for enrolment is in terms of monetary for time spent and travel.

Eligible participants will be requested for verbal and written informed consent on briefing for enrolment into the study.

All participants will be briefed, and those who participate are to adhere to;

1. No slimming medications nor slimming herbal tea are allowed for intake within the 6 month period
2. Be given a detail variety of Physical activity and dietary guidelines (Appendix G); for participants to have variety.
3. Be given on goals on dietary (in terms of calorie intake) and physical activity. A gradual decrease of intake shall be administered following the Malaysian National Dietary guidelines.

   The goals involve:

   a. Participants to calculate their current calorie intake and self regulate gradual decrease of 500 calorie intake from current intake throughout the study.
   b. Adhere to diet based on a 3 monthly calculated diet according to the activity status.
4. A custom made log book for physical activity and dietary charting, web site references for self based monitoring.

Table 6: Quick Formula for Calculating Calorie Requirement among participants for weight loss *and weight maintenance.**

Activity Status#	Overweight & Obese* (BMI > 23 kg/m^2)	Normal Weight** (BMI 18.5 – 22.9 kg/m^2)
Sedentary	20 - 25 kcal/kg	30 kcal/kg
Moderate activity	25 - 30 kcal/kg	35 kcal/kg
Marked activity	30 – 35 kcal/kg	40 kcal/kg

*Source (12). * This study utilises a cut off point of BMI>25kg/m2. ** This measure will be used when participant reached their ideal weight. To avoid multiple calculations, revised reassessment of calories requirement will be done at 3 month after study starts.*

4.8 Randomisation

Eligible participants will be randomly selected to be enrolled into the intervention or self based treatment (control) on 1:1 ratio. The randomisation will be done via a concealed envelope technique prepared by a third person not part of the research team. Number of randomisation shall be obtained using table of random numbers.

4.9 Intervention

The key element to this intervention is group social support to especially increase physical activity. Physical activity and fitness, independent of body weight have been shown to be strongly associated with cardiovascular disease risk in both diabetic and non-diabetic populations (61).

Other elements includes:

- behavioural change in terms of balanced calorie expenditure and intake by addressing both balance dietary intake and increased physical activity.

After randomisation, participants shall be grouped by members of 10-12.

A total of 16 sessions will be done within the 24 week period of intervention. One group would consist of 10-12 members and a minimum of 4/5 of the group shall be allowed to proceed with group discussion at any time (9). The group shall consist of 1 moderator / leader elected from the group member themselves and a facilitator would be made available in case of queries, to discuss the any issues pertaining to weight management.

The moderators/group leader will be given a list of problem solving topics to cover for each session. For each session, there will be three parts. The first part would involve revision of the log books for the past weeks, the 2nd part involves

discussion by the group of the issues observed in the 1st part and the 3rd part is the facilitators addressing problem solving methods.

For the overall study period of , it shall consist of 3 parts.

Part 1: Intensive 16 weeks.

This part is a knowledge transfer session from trainers to individuals between the groups and for the groups to find their dynamics.

Two/three session per day session will be held at the Perdana siswa. One session shall accommodate 2 or 3 groups. If any member missed the sessions, he/she may take other session.

Weekly exercise shall be made available for participants to take part. However, participants are encouraged to exercise at their own terms as well to achieve the physical activity goals.

Part 2: Once in 2 weeks for 8 weeks.

This session is an intermediate phase to prepare participants to practice more self management.

Part 3:Maintenance period /Follow up

After the 6 months intervention period, the centre for group meeting shall be opened for the intervention group to hold self based peer led group discussions until at end of maintenance period at 9 months post randomisation. Once in 6 weeks meeting among the facilitator and group to discuss aims and issues will be held.

4.10 Control

The control group will be briefed individually at baseline with distribution of log book on the requirement of nutritional and physical information at the beginning of the study.

This is to limit chance of meeting and create group among each other. They will continue a self based lifestyle modification throughout the 6 months period. After which they would only come for measurement of weight and blood investigations at interval 3 months and 6 months. They can however, call the 3rd person who is not part of research team to seek for clarification during the

study. Once in 3 months, a dietetic counselling will be given as part as the Wellness program given by trained dieticians.

4.11 Quality Control

A reminder system will be placed prior the interval investigation for both control and intervention group via phone calls and emails. The interval investigation would be done on separate session at 3 and 6 months of the study period.

The research team, counsellors are to audiotape each group session and discuss with the research team member for improvement.

For adherence, the intervention groups shall be followed up by calls, phone calls and emails to ensure attendance.

4.12 Rules to stop participation

Volunteers who wanted to withdraw are allowed to do so.

However, any volunteers who faced illness or symptoms of disease e.g. angina pectoris, reduced effort tolerance, depression, shall be referred for further management to their primary care physicians.

4.13 Study variables, Confounders, Operational Definitions and Scales of Measurements

The following will be the study population variables considered for this study:

4.14 Scales of Measures

Scales that will be utilised in this study are as follows. Each of the measurement tools will be calibrated and validated before use.

i. Weighing scale – measured to the nearest 0.1 kilogram with a digital scale SECA model HD 309.
ii. Height measurement – measures to the precision of 0.05cm with Body meter SECA Model 208.
iii. Waist circumference –measures to 0.01 nearest cm, using non elastic SECA measuring tape.
iv. Sphygmomanometer –OMRON digital

Item i-iv, will be taken twice and average out.

v. Laboratory machines

a. Lipid profile (mg/dl)

b. Fasting Blood Glucose (mg/cm3)

4.15 Methods of data collection

Questionnaire based data are as in Table below.

Table 7: Summary of Self Administered questionnaire to be used

Outcome measures	Tools / Equipments	Specifics	Reliability	Validity
Dietary, kcal	Questionnaire	24hr call-recall<br>7 day dietary log	Established	+
Physical activity	Questionnaire	Short form IPAQ	Established	+
Self efficacy	Questionnaire	Weight Efficacy Lifestyle, WEL	Internal consistency of 0.7-0.9, of 5 subscales (Clark et al, Pamela et al & Navidian A et al)	+
Perceived Social Support	Questionnaire	Multi dimensional perceived social support (Zimmet 1988)	Has been used in obesity study (Chang et al)	+
Automatic thoughts	Questionnaire	Negative thoughts	Has been used in local for assessment of ATQ (Mukhtar F et al)	+

4.15.2 Continuous

Clinical parameter e.g. Blood pressure, weight, height, Fasting Serum Lipids, and Fasting Blood glucose.

It shall be collected by: 2 medical staff employed part time for the purpose of this research by the researcher. This is to have at least 1 side blinded in this study in effort to reduce bias in measurement.

a) Physical examination

i) Weight

For each session of measurement (Interval 0, 3, 6 and 9 months), each participants will be weighed and measured twice by 2 different personnel. This measurement will be average. The participants will be required to stand without shoes.

ii) Height

The participants are required to stand bare feet against a straight wall below the centre of measuring tongue of the body meter, lean against the wall with head facing straight, the back of the head (occipital region), shoulder, buttock and heels against the wall, hand loosely on the side and heels resting together. The measuring tongue will be lowered until it gently touched the top of the head.

iii) Waist Circumference

The waist circumference will be measured at midpoint between the inferior margin of the last rib and the crest of the ilium.

iv) Blood pressure

Blood pressure monitoring would require participants to relax for 10-15 minutes within arrival. It shall follow the Clinical Practice Guidelines for management of Hypertension 3rd edition on methods of blood pressure monitoring.Blood pressure should be measured in both arms and the higher reading is taken as the systemic BP.

b) Blood investigations

i) Fasting serum cholesterol

Participants are required to fast 8 hours prior to blood taking to assess on the Serum cholesterol level. Blood taking (venepuncture) will be performed in the wellness clinic. The blood will be collected in the wellness clinic stored and test in batches by the lab technician

ii) Fasting Blood Sugar

Venepuncture will be performed in the Wellness clinic. The blood will be collected in the Wellness clinic stored and sent to a UMMC.

4.16. Plan for data collection and processing

4.16.1 Data collection plan

In general, the data will be collected from the period of randomisation to the end of study at the 6th month period and at 9 month

Specific timeframe for data collection are as below.

Table 8: Data collection timeframe and validation

	Type of data	Time frame for data collection	Validation
i.	Sociodemographic data	Distribution of the demographic self reported questionnaire shall be done on the day of registration.	Verification of data provided against the identification card will be done on the registration day.
ii.	24 hour diet call recall for dietary intake		Data verification checked against the food diary.
iii.	Short IPAQ Physical activity	At baseline<br>6 months (post intervention) and,<br>9 months (post maintenance)	Data verification checked against the PA diary.
iv.	Weight efficacy Lifestyle,<br>Self efficacy		Checked for completion on submission at the timed interval and request for immediate fill up.
v.	Multidimensional Perceived Social Support		
vi.	Automatic negative thoughts		Checked for completion on submission at the timed interval and request for immediate fill up.
vii.	Weight, height^{+} and blood pressure	Baseline, 3, 6 and 9 months	Immediate verification of results will be done by two trained health personnel.
viii.	Blood parameters; Cholesterol<br>HbA1c	These samples will be taken by the 3rd party staff in the wellness clinic at the 0, 3 and 6 months study period.<br>Then process by the UMMC lab staffs.	Verification shall be done by in accordance to the lab audit trail prepared by the UMMC lab.

$^{+}$ only taken at baseline.

Verified data are re checked on situ for questionnaire. Missing data on the questionnaire will be requested to be filled up on site or subsequent session.

4.17 Plan for data analysis

Collected data will be entered as obtained. Data cleaning and identification of outliers will be rechecked and identified. Double entry shall be done. The analysis of data will utilize Statistical Package for Social Science, (SPSS) version 16. Missing data are to be ensured unable to be collected before filling up using last observation carried forward in repeated measure data, or mean in demographic data. Frequency distribution table and descriptive statistics (standard deviation and means) will be run to observe the nature of the sample obtained. Codes from the questionnaire form will be computed into the SPSS for changing of coding and categorising. This will be done for continuous data e.g. age income and clinical outcome i.e. Blood pressure, BMI, Fasting serum lipids and fasting blood glucose.

4.18 Record Keeping

All the records of research shall be kept via hard and soft copy in duplicates. A period of minimum 7 years shall be made for the hard copy.

4.19 Pre test and validation of study instrument / Pilot

A pilot run will be done before the 1 month recruitment period in terms logistic and others in the wellness clinic in June 2011. The intent is to improvise the study design, logistics, flow and management of the study.

5. LIMITATIONS OF STUDY AND MEASURES TO LIMIT THEM

5.1 Main Limitations

This study is limited by factors associated with the design itself and financial and time constraint.

5.1.1 Blinding

Community based randomised control trial has constraint in ability to do blinding of both participants and investigator. This would result in bias that would affect the study outcome. Blinding on the researcher and participants is not possible as researcher is part of the provider that overlooks the overall progress of the study and participants are obvious to what treatment they are allocated to. Furthermore in this study as it is done in community setting which it is less controlled.

Concealment in randomisation is done by a third person who is not part of the research team.

5.1.2 Contamination/ crossover of groups

In a community set up cross over may occur as participants may meet and exchange information and create their own groups outside the programme especially in the individual therapy group. This is minimised by providing the intervention separately and minimised encounter.

5.1.3 Weight maintenance and follow up

Long term follow up to observe for weight maintenance is required to observe the true effect of group support. However, in constraint of the study period and financial means. This study is maintained at 6 months period for intervention phase with a 3 months follow up period to observe for maintenance.

6. ETHICAL CONSIDERATIONS

This RCT required approval from the ethical board in the University Malaya.

This in particular so as there is direct handling of community that will be subjected to intervention in terms of:

- Screening
- Questionnaire
- Blood sampling
- Lifestyle modification

The flyers and banners content would request approval from the Ethic committee.

Participants will be informed of the availability of group support as being the intervention tool. And that they would be assigned randomly. Though they are not encouraged to change group during the study they are by all means are to withdraw from the study.

They shall be informed though they withdrawn they will still be monitored as part as the intention to treat role of the study. Verbal and written informed consent would be obtained on agreement to be enrolled in the study.

Participants would be ensured of confidentiality as they participate in this study.

WORKPLAN (GANTT CHART)

WORKPLAN PROPOSAL (GANTT CHART)

No	Activities	Period (weeks)	Starts	End	October	Nov 2010	Dec 2010	Jan 2011	Feb 2011	March	April 2011	May	Jun 2011	July 2011	August	Sept 2011	October	Nov 2011	Dec 2011	Jan 2012	Feb 2012	March 2012	April 2012	May 2012	Jun 2012	July 2012	August	Sept 2012	October 2012	Nov 2012	Dec 2012
1.	SUBMISSION TO ETHICS COMMITTEE																														
	Submission to ethic committee (approval and funding)			Feb 2010						X	X																				
2.	RESEARCH IMPEMENTATION																														
a.	Screening	12	5/2011	7/2011								X	X	X																	
b.	Recruitment	16	5/2011	8/2011								X	X	X	X																
c.	Intervention Phase 1a	12	1/9/11	1/11/11												X	X	X													
d.	Intervention Phase1b	12	12/11	2/12															X	X	X										
e.	Intervention Phase 2	12	3/12	5/12																		X	X	X							

Reference

1. Colin Mathers GSaMM, WHO. Global Health Risk2008.
2. Dixon JB. The effect of obesity on health outcomes. Molecular and Cellular Endocrinology. [doi: DOI: 10.1016/j.mce.2009.07.008]. 2010;316(2):104-8.
3. Obesity and overweight [database on the Internet]. WHO Media Centre. 2006 [cited Jan 2010]. Available from: http://www.who.int/mediacentre/factsheets/fs311/en/.
4. Caballero B. The Global Epidemic of Obesity: An Overview. Epidemiologic Reviews2007 January 1, 2007;29(1):1-5.
5. Malaysia MoH. National Health Morbidity Survey III. 2006.
6. Seen KAZaLS. Trends in overweight and obese adults in Malaysia (1996–2009): a systematic review. Obesity Reviews 2010;11:403-12.
7. Health Education Division MoH, Malaysia. Healthy Lifestyle Campaign. 2005 [cited 2010 10th March]; Available from: http://www.infosihat.gov.my/kchs/KCHS%202005%20-%20Setting%20Tempat%20Kerja/KCHS%202005.php.
8. Boutayeb A. The double burden of communicable and non-communicable diseases in developing countries. Transactions of the Royal Society of Tropical Medicine and Hygiene. [doi: DOI: 10.1016/j.trstmh.2005.07.021]. 2006;100(3):191-9.
9. Wadden TA, Butryn ML, Wilson C. Lifestyle Modification for the Management of Obesity. Gastroenterology. [doi: DOI: 10.1053/j.gastro.2007.03.051]. 2007;132(6):2226-38.
10. Chang CT. Applicability of the stages of change and Weight Efficacy Lifestyle Questionnaire with natives of Sarawak, Malaysia. INternational Electronic Journal of Rural and Remote Health research, Education, Practice and Policy 2007;2007(864).
11. Kong W-T, Chua S-S, Alwi S. Weight Loss Practices Among Malaysian Adults. Asia-Pacific Journal of Public Health2002 July 1, 2002;14(2):99-104.
12. Ministry of Health M, Committee. Clinical Practice Guidelines. Obesity 2004.
13. Pedro J Teixeira* ALP, Teresa L Branco, Sandra S Martins, Cláudia S Minderico, José T Barata, Analiza M Silva and Luís B Sardinha. Who will lose weight? A reexamination of predictors of weight loss in women. International Journal of Behavioral Nutrition and Physical Activity 2004; 1 (12).
14. Fabricatore AN, Wadden TA, Moore RH, Butryn ML, Heymsfield SB, Nguyen AM. Predictors of attrition and weight loss success: Results from a randomized controlled trial. Behaviour Research and Therapy2009;47(8):685-91.
15. Edell BH, Edington S, Herd B, O'Brien RM, Witkin G. Self-efficacy and self-motivation as predictors of weight loss. Addictive Behaviors1987;12(1):63-6.
16. Saito H, Kimura Y, Tashima S, Takao N, Nakagawa A, Baba T, et al. Psychological factors that promote behavior modification by obese patients. BioPsychoSocial Medicine2009;3(1):9.
17. Levy RL, Finch EA, Crowell MD, Talley NJ, Jeffery RW. Behavioral intervention for the treatment of obesity: strategies and effectiveness data. The American Journal Of Gastroenterology2007;102(10):2314-21.
18. Gallant MP. The Influence of Social Support on Chronic Illness Self-Management: A Review and Directions for Research. Health Education & Behavior2003 April 1, 2003;30(2):170-95.
19. Renjilian DA, Perri MG, Nezu AM, McKelvey WF, Shermer RL, Anton SD. Individual versus group therapy for obesity: Effects of matching participants to their treatment preferences. Journal of Consulting and Clinical Psychology2001;69(4):717-21.
20. Waleekhachonloet O-A, Limwattananon C, Limwattananon S, Gross CR. Group behavior therapy versus individual behavior therapy for healthy dieting and weight control management in overweight and obese women living in rural community. Obesity Research & Clinical Practice. [doi: DOI: 10.1016/j.orcp.2007.07.005]. 2007;1(4):223-32.
21. Coker RJ. Public Health Impact of Detention of individuals with tuberculosis; systematic literature review. Journal of The Royal Institute of Public Health2002;2003(117):281-7.
22. Rampal S, Rampal L, Rahmat R, Azhar Md Zain, Yee Guan Yap, Mohamed M, et al. Variation in the Prevalence, Awareness, and Control of Diabetes in a Multiethnic Population: A Nationwide Population Study in Malaysia. Asia-Pacific Journal of Public Health2010 April 1, 2010;22(2):194-202.

23. Rampal L, Rampal S, Azhar MZ, Rahman AR. Prevalence, awareness, treatment and control of hypertension in Malaysia: A national study of 16,440 subjects. Public Health2008;122(1):11-8.
24. Rampal L, Rampal S, Khor GL, Zain AM, Ooyub S, Rahmat R, et al. A National Study on the Prevalence of OBesity Among 16,127 Malaysians. Asia Pacific Jpurnal of Public Health2007;16(3):561.
25. Sidik SM, Rampal L. The Prevalence and factors associated with obesity among adult women in Selangor, Malaysia. Asia Pacific Family Medicine2009;2009(8):6.
26. Flint AJ, Hu FB, Glynn RJ, Caspard H, Manson JE, Willett WC, et al. Excess Weight and the Risk of Incident Coronary Heart Disease Among Men and Women. Obesity2009;18(2):377-83.
27. Bellver J, Ayllón Y, Ferrando M, Melo M, Goyri E, Pellicer A, et al. Female obesity impairs in vitro fertilization outcome without affecting embryo quality. Fertility and Sterility. [doi: DOI: 10.1016/j.fertnstert.2008.12.032]. 2010;93(2):447-54.
28. Peeters A, Bonneux L, Nusselder WJ, De Laet C, Barendregt JJ. Adult Obesity and the Burden of Disability throughout Life[ast][ast]. Obesity2004;12(7):1145-51.
29. Kenneth F. Adams PD, Arthur Schatzkin, M.D., Tamara B. Harris, M.D., Victor Kipnis, Ph.D.,Traci Mouw, M.P.H., Rachel Ballard-Barbash, M.D., Albert Hollenbeck, Ph.D., and Michael F. Leitzmann, M.D. Overweight, Obesity, and Mortality in a Large Prospective Cohort of Persons 50 to 71 Years Old. New England Journal of Medicine2006;355(8):763-78.
30. Maciejewski ML, Patrick DL, Williamson DF. A structured review of randomized controlled trials of weight loss showed little improvement in health-related quality of life. Journal of Clinical Epidemiology2005;58(6):568-78.
31. Fatimah A MIM, Romzi MA and Faizah H. Perception of bodyweight status among office workers in two government departments in Kuala Lumpur. Malaysian Journal of Nutrition1995;1(1):11-9.
32. Moy FM, Sallam AA, Wong ML. Dietary Modification in a Workplace Health Promotion Programme in Kuala Lumpur, Malaysia. Asia Pacific Jpurnal of Public Health2006;20(October 2008):5.
33. Suriati W, editor. Promoting Weight Loss among Obese Participant Community-based Behavioral Intervention Program in Kelantan. MASO Scientific Conference Symposium; 2009.
34. Norawai A. Community Lifestyle Intervention Program on Obesity Control. Malaysian Family Physician2009;4 (Supplement 1).
35. Romero-Corral A, Somers VK, Sierra-Johnson J, Thomas RJ, Collazo-Clavell ML, Korinek J, et al. Accuracy of body mass index in diagnosing obesity in the adult general population. Int J Obes2008;32(6):959-66.
36. Deduja V, Misra A, Pandey RM, G.Devina, Kumar G, Vikram NK. BMI does not accurately predict overweight in Asian Indians in Northern India. British Journal of Nutrition2001;2001(86):105-12.
37. Janssen I, Katzmarzyk PT, Ross R. Waist circumference and not body mass index explains obesity-related health risk. Am J Clin Nutr2004 March 1, 2004;79(3):379-84.
38. Fuentes. Effect of moderate diet induced weight loss and weight regain on cardiovascular structure and function. 2009.
39. Rickheim PL, Flader JL, Weaver TW, Kendall DM. Assessment of group versus individual diabetes education - A randomized study. Diabetes Care2002 Feb;25(2):269-74.
40. Thomas A Wadden dSW, Rebecca H. Meiberg, Rena R Wing, Donna H Ryan, Karen C Johnson, John P. Forrey, Dace L.Trence and Mara Z. Vitolins. One-year Weight Losses in the Look AHEAD Study: Factors Associated With Success. Obesity Journal2009;2009(17):713-22.
41. Sartorelli DS, Franco LJ, Cardoso MA. High intake of fruits and vegetables predicts weight loss in Brazilian overweight adults. Nutrition Research2008;28(4):233-8.
42. Knowler WC, Barrett-Connor E, Fowler SE, Hamman RF, Lachin JM, Walker EA, et al. Reduction in the incidence of type 2 diabetes with lifestyle intervention or metformin. The New England Journal Of Medicine2002;346(6):393-403.
43. Wadden TA, Foster GD. Behavioral treatment of obesity. Med Clin North Am2000 Mar;84(2):441-61, vii.
44. Foster GD, Makris AP, Bailer BA. Behavioral treatment of obesity. Am J Clin Nutr2005 July 1, 2005;82(1):230S-5.

45. Maija Reblin MaBNU, PhD. Social and Emotional Support and its Implication for Health. Curr Opin Psychiatry2008;2008 March (21(2)):201-5.
46. Davidson KP, Pennebaker JW, Dickerson SS. Who Talks? Social Psychology of Illness Support Groups. American Psychologist2000;55(2):205-17.
47. L.F S, T L. Group Behaviour Therapy Programmes for Smoking Cessation (Review). Cohrane Collaboration2006;2006(1).
48. Dam HAv, Horst FGvd, Knoops L, Rykman RM, Crebolder HFJM, Borne BHWvd. Social Support in Diabetes: A systematic review of controlled intervention studies. Patient Education and Counseling2005;59(2005):1-12.
49. Verheijden MW, Bakx JC, van Weel C, Koelen MA, van Staveren WA. Role of social support in lifestyle-focused weight management interventions. Eur J Clin Nutr;59(S1):S179-S86.
50. Smith BK, Walleghen ELV, Wiens GC, Martin RN, Curry CR, SUllivan DK, et al. Comparison of two self directed weight loss intervention: LImited weekly support vs no outside support. Asian Oceanian Association for The Study of Obesity2009;2003(3):149-57.
51. Clark MM, Abrams DB, Niaura RS, Eaton CA, Rossi JS. Self-efficacy in weight management. Journal of Consulting and Clinical Psychology1991;59(5):739-44.
52. Zimet GD, Powell SS, Farley GK, Werkman S, Berkoff KA. Psychometric characteristics of the Multidimensional Scale of Perceived Social Support. Journal Of Personality Assessment1990 1990 Winter;55(3-4):610-7.
53. Wing RR, Tate DF, Gorin AA, Raynor HA, Fava JL. A Self-Regulation Program for Maintenance of Weight Loss. New England Journal of Medicine2006;355(15):1563-71.
54. Ash S, Reeves M, Bauer J, Dover T, Vivanti A, Leong C, et al. A randomised control trial comparing lifestyle groups, individual counselling and written information in the management of weight and health outcomes over 12 months. Int J Obes2006;30(10):1557-64.
55. Pettman TL, Buckley JD, Misan GMH, Coates AM, Howe PRC. Health benefits of a 4-month group-based diet and lifestyle modification program for individuals with metabolic syndrome. Obesity Research & Clinical Practice2009;3(4):221-35.
56. McConnon A, Kirk SF, Cockroft J, Harvey EL, Greenwood DC, Thomas JD, et al. The Internet for Weight Control in An Obese Sample: Results of A Randomised Controlled Trial. BioMed Central Health Service Research2007;2007(7):9.
57. Jessica Goke-LaRose AAG, Rena R Wing. Behaviour Self Regulation for Weight Loss In Young Adults: A randomised Control Trial. International Journal of Behavioral Nutrition and Physical Activity2009;2009:1-9.
58. Christie A. Befort a, Joseph E. Donnelly b, Debra K. Sullivan c, Edward F. Ellerbeck a, Michael G. Perri d. Group versus individual phone-based obesity treatment for rural women. Eating Behaviors2009;11((2010)):11-7.
59. Aine McConnon SFK, Jennie E Crockroft, Emma L Harvey, darren C Greenwood, James D Thomas, Joan K. Ransley, Laura Bojke. The Internet for Weight Control in an Obese Sample: A randomised controlled trial. BMC Health Service Research20007;December.
60. Pettman TL MG, Owen K, Warren K, Coates AM, Buckley JD, Howe PR. Self-management for obesity and cardio-metabolic fitness: Description and evaluation of the lifestyle modification program of a randomised controlled trial. Int J Behav Nutr Phys Act2008 Oct 27(5):53.
61. Committee LAPR. Protocol Action for Health in Diabetes Look AHEAD Clinical TrialApril 29, 2009.
62. Michael G. Perri PMCL, MD; Patricia E. Durning, PhD; David M. Janicke, PhD;. Extended Care Programme for weight management in rural communities (TOURS). Arch Internal Medicine2008;168(21):2347-54.
63. Consultation. WE. Appropriate body-mass index for Asian populations and its implications for policy and intervention strategies. Lancet 2004;Jan 10 2004(363(9403)):157-63.
64. Stevens J, Taber DR, Murray DM, Ward DS. Advances and Controversies in the Design of Obesity Prevention Trials[ast]. Obesity2007;15(9):2163-70.

Appendix 1

Appendix 1- Map

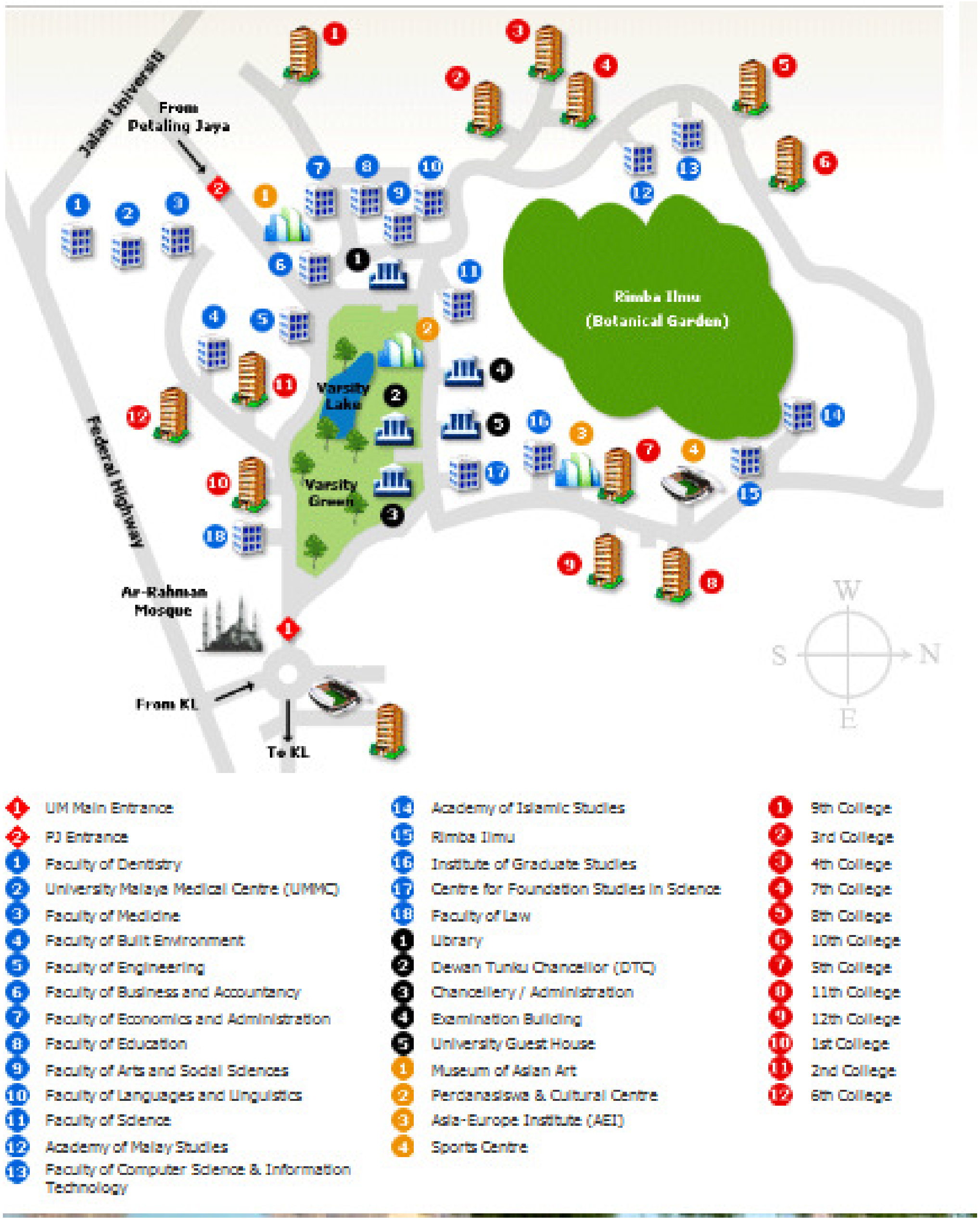

The main campus of University Malaya located in Petaling Jaya, Kuala Lumpur spread across 922 acre land.

Appendix 2

Appendix 2 – Flow Chart

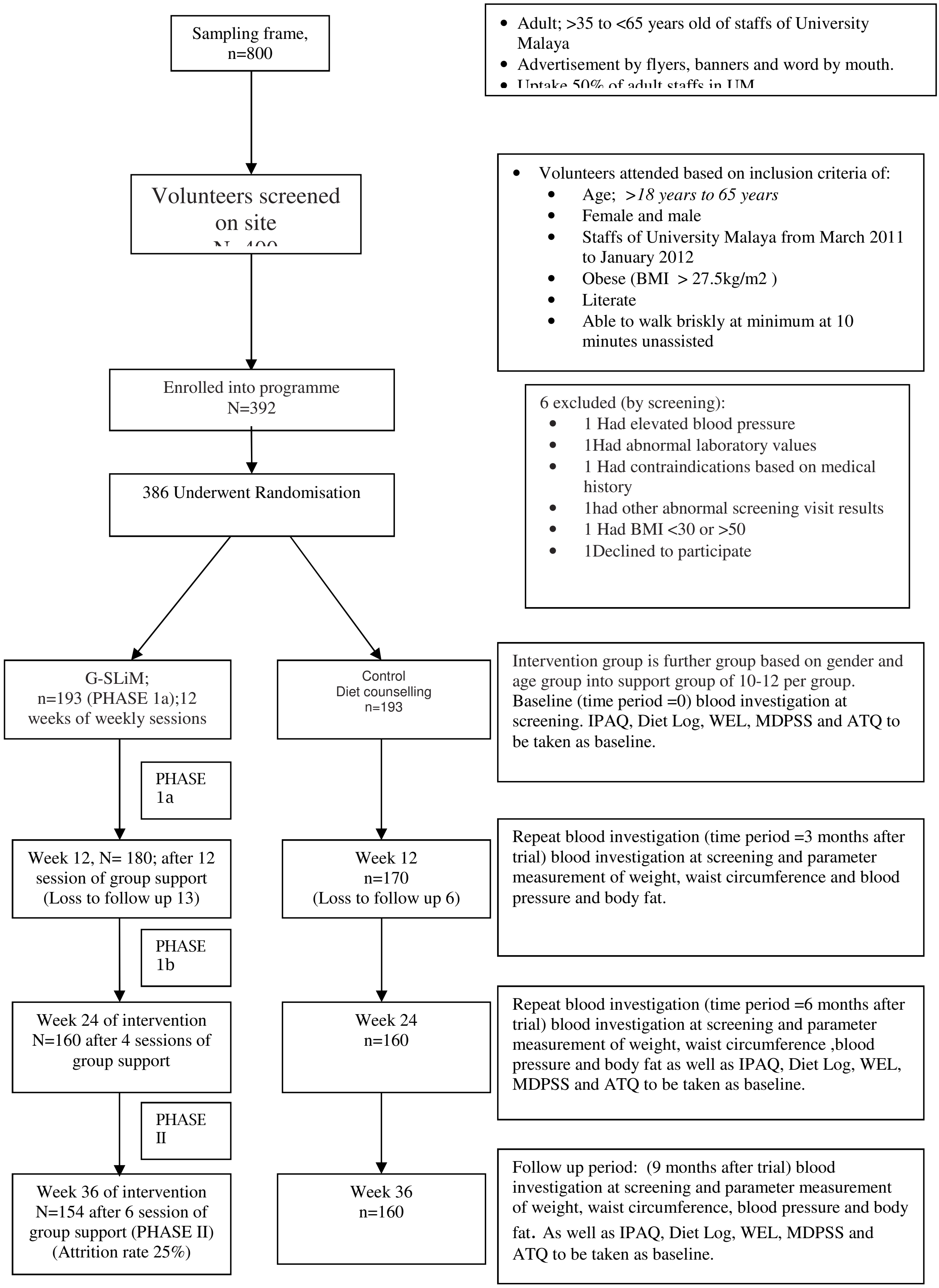

Supplement: S1 File — (PDF) [file pone.0160343.s001.pdf]
