# Supplementary material for: Effectiveness of a Group Support Lifestyle Modification (GSLiM) Programme among Obese Adults in Workplace: A Randomised Controlled Trial
Source: PLoS One. 2016 Aug 18;11(8):e0160343. doi: 10.1371/journal.pone.0160343 (PMC4990415; doi:10.1371/journal.pone.0160343)
Supplement: S1 Table — (PDF) [file pone.0160343.s003.pdf]

# PONE – D- 16-06104; Effectiveness of a Group Support Lifestyle Modification (GSLiM) Programme among Obese Adult in Workplace: A Randomised Controlled Trial
# CONSORT 2010 checklist of information to include when reporting a randomised trial

| Section/Topic | Item No | Checklist item | Reported on page No |
|---|---|---|---|
| **Title and abstract** | | | |
| | 1a | Identification as a randomised trial in the title | line 1 – 3; page 1 |
| | 1b | Structured summary of trial design, methods, results, and conclusions (for specific guidance see CONSORT for abstracts) | Page 2 |
| **Introduction** | | | |
| Background and objectives | 2a | Scientific background and explanation of rationale | line 50 – 77; page 4 - 5 |
| | 2b | Specific objectives or hypotheses | line 80 to 85; page 5 |
| **Methods** | | | |
| Trial design | 3a | Description of trial design (such as parallel, factorial) including allocation ratio | line 87; page 5 |
| | 3b | Important changes to methods after trial commencement (such as eligibility criteria), with reasons | Nil |
| Participants | 4a | Eligibility criteria for participants | line 99 – 103; page 6 |
| | 4b | Settings and locations where the data were collected | line 97 - 98; page 6 |
| Interventions | 5 | The interventions for each group with sufficient details to allow replication, including how and when they were actually administered | Line 126 – 170; page 11 - 12 |
| Outcomes | 6a | Completely defined pre-specified primary and secondary outcome measures, including how and when they were assessed | Line 172 – 220; page 13 -14 |
| | 6b | Any changes to trial outcomes after the trial commenced, with reasons | Nil |
| Sample size | 7a | How sample size was determined | Line 222- 225; page 14 |
| | 7b | When applicable, explanation of any interim analyses and stopping guidelines | Nil |
| Randomisation: | | | |
| Sequence | 8a | Method used to generate the random allocation sequence | Line 116 - 117; |

| | | | |
|---|---|---|---|
| generation | | | page 7 |
| | 8b | Type of randomisation; details of any restriction (such as blocking and block size) | Line 115; page 7 |
| Allocation concealment mechanism | 9 | Mechanism used to implement the random allocation sequence (such as sequentially numbered containers), describing any steps taken to conceal the sequence until interventions were assigned | Line 117 - 118; page 7 |
| Implementation | 10 | Who generated the random allocation sequence, who enrolled participants, and who assigned participants to interventions | Line 114, page 7 |
| Blinding | 11a | If done, who was blinded after assignment to interventions (for example, participants, care providers, those assessing outcomes) and how | Line 117 - 118; page 7 |
| | 11b | If relevant, description of the similarity of interventions | - |
| Statistical methods | 12a | Statistical methods used to compare groups for primary and secondary outcomes | Line 237 – 238; page 15 |
| | 12b | Methods for additional analyses, such as subgroup analyses and adjusted analyses | Line 241 – 243; Page 15 |
| **Results** | | | |
| Participant flow (a diagram is strongly recommended) | 13a | For each group, the numbers of participants who were randomly assigned, received intended treatment, and were analysed for the primary outcome | Flow diagram |
| | 13b | For each group, losses and exclusions after randomisation, together with reasons | Flow diagram |
| Recruitment | 14a | Dates defining the periods of recruitment and follow-up | Line 94-95; page 6 |
| | 14b | Why the trial ended or was stopped | - |
| Baseline data | 15 | A table showing baseline demographic and clinical characteristics for each group | Table 4; Page 17 |
| Numbers analysed | 16 | For each group, number of participants (denominator) included in each analysis and whether the analysis was by original assigned groups | Table 4; Page 17 |
| Outcomes and estimation | 17a | For each primary and secondary outcome, results for each group, and the estimated effect size and its precision (such as 95% confidence interval) | Table 6 -8 ; page 19 - 22 |
| | 17b | For binary outcomes, presentation of both absolute and relative effect sizes is recommended | Line 261 – 262 , page 18; |
| Ancillary analyses | 18 | Results of any other analyses performed, including subgroup analyses and adjusted analyses, distinguishing pre-specified from exploratory | - |
| Harms | 19 | All important harms or unintended effects in each group (for specific guidance see CONSORT for harms) | Line 350- 253; |

| | | | |
|---|---|---|---|
| | | | Page 24 |
| **Discussion** | | | |
| Limitations | 20 | Trial limitations, addressing sources of potential bias, imprecision, and, if relevant, multiplicity of analyses | Line 418 – 444; page 28 - 29 |
| Generalisability | 21 | Generalisability (external validity, applicability) of the trial findings | Line 415 – 417 ; page 28 |
| Interpretation | 22 | Interpretation consistent with results, balancing benefits and harms, and considering other relevant evidence | Line 370 – 414; Page 24 -25 |
| **Other information** | | | |
| Registration | 23 | Registration number and name of trial registry | Line 88 – 89; Page 5 |
| Protocol | 24 | Where the full trial protocol can be accessed, if available | With this submission as additional information |
| Funding | 25 | Sources of funding and other support (such as supply of drugs), role of funders | Line 90; Page 5 |
